# Characterization of a metabolomic profile associated with responsiveness to therapy in the acute phase of septic shock

**DOI:** 10.1038/s41598-017-09619-x

**Published:** 2017-08-29

**Authors:** Alice Cambiaghi, Bernardo Bollen Pinto, Laura Brunelli, Francesca Falcetta, Federico Aletti, Karim Bendjelid, Roberta Pastorelli, Manuela Ferrario

**Affiliations:** 1 Politecnico di Milano, Milan, Italy; 2 Hôpitaux Universitaires de Genève, Genève, Switzerland; 3 IRCCS-Istituto di Ricerche Farmacologiche Mario Negri, Milan, Italy

## Abstract

The early metabolic signatures associated with the progression of septic shock and with responsiveness to therapy can be useful for developing target therapy. The Sequential Organ Failure Assessment (SOFA) score is used for stratifying risk and predicting mortality. This study aimed to verify whether different responses to therapy, assessed as changes in SOFA score at admission (T1, acute phase) and 48 h later (T2, post-resuscitation), are associated with different metabolite patterns. We examined the plasma metabolome of 21 septic shock patients (pts) enrolled in the Shockomics clinical trial (NCT02141607). Patients for which SOFA_T2_ was >8 and Δ = SOFA_T1_ − SOFA_T2_ < 5, were classified as not responsive to therapy (NR, 7 pts), the remaining 14 as responsive (R). We combined untargeted and targeted mass spectrometry-based metabolomics strategies to cover the plasma metabolites repertoire as far as possible. Metabolite concentration changes from T1 to T2 (Δ = T2 − T1) were used to build classification models. Our results support the emerging evidence that lipidome alterations play an important role in individual patients’ responses to infection. Furthermore, alanine indicates a possible alteration in the glucose-alanine cycle in the liver, providing a different picture of liver functionality from bilirubin. Understanding these metabolic disturbances is important for developing any effective tailored therapy for these patients.

## Introduction

Sepsis is a life-threatening organ dysfunction caused by a dysregulated host response to infection^1^. Septic shock is a subset of sepsis with underlying circulatory and cellular/metabolic abnormalities associated with higher mortality rates^1, 2^.

Early supportive therapy with fluid resuscitation and vasopressors to restore hemodynamics and reduce tissue hypoperfusion is decisive for the patient’s outcome and has figured in treatment guidelines for decades^3^. However, mortality rates for septic shock may reach 60% even in the era of early recognition and treatment^4^, with today’s poor prognosis mainly related to multi-organ dysfunction (MOF).

The limited improvement in septic shock survival can be explained by the inability to prospectively identify the patients who are most likely to benefit from a specific therapy, and the lack of predictive monitoring markers of drug delivery and response. Researchers are becoming increasingly aware that the response to therapy is crucial and precision medicine is already an important research topic for acute illnesses and septic shock^5^. Precision medicine extends personalized medicine beyond the genome to include broader systems, with a multilevel approach to tailoring therapeutics to individual patients.

Recently interest in the metabolomics approach has grown as the metabolome is the result of gene and protein function and activity and may therefore provide a more sensitive readout of drug response phenotypes because most drugs affect components of metabolism^6^. For instance, metabolomics analysis of various classes of blood metabolites has been used to search for predictive signatures of intensive care unit (ICU) mortality in adults^7–11^. Less attention has been paid to the investigation of putative metabolic determinants that would able to classify patient’s responsiveness to initial therapy during the first 48 h in ICU.

We investigated the plasma metabolomics profile of septic shock patients during the acute phase of resuscitation. Blood samples were collected at study enrolment (time T1) and after about 48 hours (time T2). The patients received initial therapy according to the standards^3^ immediately after shock diagnosis (time T0). The time interval between T0 and T1 was on average 10 hours.

We merged untargeted and targeted mass spectrometry-based metabolomics strategies to cover the plasma metabolites repertoire as far as possible. We first adopted an unbiased strategy (untargeted metabolomics), profiling as many plasma metabolites as possible without any *a-priori* hypothesis. We applied rapid but yet accurate mass metabolic profiling by direct flow injection-time-of-flight mass spectrometry (TOF-MS)^12^ as untargeted screening to explore the main perturbed metabolic features.

Targeted metabolomics measures and quantifies a specific list of metabolites according to a standard in order to achieve absolute quantification of defined metabolite classes^13^. Since metabolic signatures showing changes in circulating kynurenine, fatty acids, lysophosphatidylcholines species and/or carnitine esters have already been reported in different septic shock^14–18^, they might conceivably be involved in the first phase of shock as well, and could help in understanding the different trajectories in septic shock patients. We therefore also applied a targeted approach focused on measuring these specific metabolic classes to provide the magnitude of their changes in our clinical setting and possibly validate the information from our untargeted analysis.

The primary objective was to verify whether different responses to therapy, measured as changes in organ dysfunction using the Sequential Organ Failure Assessment (SOFA) score, are associated with different metabolite patterns.

## Results

### Clinical characteristics of the study population

The current work is an ancillary study from the multicenter prospective observational trial, Shockomics (see ClinicalTrials.gov Identifier NCT02141607). We analyzed the septic shock patients enrolled at the Université de Gèneve (Geneva, Switzerland).

All patients had a SOFA score higher or equal than 9 at T1 (Table 1). Patients who still had a score higher than 8 at T2 and had no decrease of at least 4 points were classified as not responsive to therapy (NR); the other cases were classified as responsive to therapy (R). The NR group consisted of seven patients who had a SOFA score >8 at T2 and Δ SOFA <5 (Δ = T1 − T2 values of SOFA).

**Table 1:** Characteristics of the two groups of patients at study enrollment (R: responsive; NR: not responsive to therapy).

	All Patients	R	NR
no patients	21	12 (57%)	9 (43%)
Sex (Male) [no (%)]	16 (76%)	8 (67%)	8 (89%)
Weight (kg)	85 (73.75, 90.5)	82.5 (72.5, 90.5)	85 (76.75, 94)
BMI - *Body Mass Index*	26.85 (24.975, 30.335)	26.54 (23.505, 30.630)	27.76 (25.46, 30.407)
Age (years)	69.649 (63.366, 80.26)	66.503 (60.622, 74.784)	74.918 (66.198, 81.886)
No Affected Organs	4 (3, 5)	4 (3.5, 5)	4 (3, 5)
Lactate (mmol/L)	4 (2.95, 5.6)	4.25 (3, 5.6)	3.6 (2.7, 5.275)
Systolic Arterial Pressure (mmHg)	85 (74.5, 91.25)	87.5 (74, 91.5)	85 (80.25, 91.25)
Diastolic Arterial Pressure (mmHg)	47 (44.5, 49.5)	46.5 (42.5, 47)	49 (46.5, 51.25)
Mean Arterial Pressure (mmHg)	60 (56.25, 62.25)	58.5 (54, 61.5)	62 (58.5, 63.25)
Heart Rate (bpm)	111 (91.5, 125.5)	110 (89.5, 122)	111 (98, 134)
Respiratory Rate (breath per minute)	20 (19, 25.5)	20.5 (18, 25.5)	20 (19.75, 26.25)
GCS	3 (3, 5.25)	3 (3, 4.5)	3 (3, 6)
SAS	2 (1, 2.25)	2 (1, 2.5)	1 (1, 2.25)
SOFA	14 (12, 15.25)	14 (13, 15.5)	13 (10.75, 15.25)
APACHE-II	34 (29.75, 38)	35 (31, 37.5)	31 (28.5, 41)
Temperature (°C)	37.5 (36.75, 38.05)	37.45 (36.7, 37.9)	37.5 (36.975, 38.6)
Urine Output (mL/day)	1550 (803.75, 1945)	1778.5 (1135, 1975)	805 (417.5, 1741.25)
Hematocrit (%)	35 (30.65, 38)	35.05 (31.8, 36.85)	35 (29.95, 41.75)
White Blood Cells (10^3^/mm^3^)	11.7 (9.75, 19.675)	14.3 (10.15, 27.55)	10.5 (9.2, 12.6)
Creatinine (mg/dL)	1.7 (1.175, 2.025)	1.65 (1.3, 2.05)	1.7 (1.075, 2.075)
Na (mmol/L)	140 (135, 140.25)	140 (136.5, 144.5)	139 (133, 140)
K (mmol/L)	4.2 (3.9, 4.725)	4.2 (3.55, 4.85)	4.3 (4.15, 4.725)
Platelets (10^3^/mm^3^)	168 (95.5, 198)	153 (90, 197)	194 (94.5, 202.25)
Biliuribine (mg/dL)	1.3 (0.95, 2.05)	1.44 (1.025, 1.95)	1.3 (0.775, 3.7)
Glycemia (mg/dL)	167 (124.25, 196.5)	160 (120.5, 178)	185 (119.5, 212.5)
Prothrombin Time INR	1.3 (1.1, 1.5)	1.4 (1.15, 1.55)	1.2 (1.1, 1.325)
Chloride (mEq/L)	106 (101.25, 109.25)	108.5 (103.5, 113)	103 (97, 106)
aPPT - activated Partial Thromboplastin Time	46.3 (42.525, 58.175)	47.3 (42.25, 60.2)	46.1 (42.9, 56.85)
Prothrombin Time (seconds)	62 (49.25, 81.25)	59 (41.5, 76)	62 (56.5, 86)
Fibrinogen (mg/dL)	4.7 (3.95, 6.625)	4.8 (4.6, 6.25)	4.5 (2.275, 7.325)
C-Reactive Protein (mg/L)	273.4 (155.4, 352.05)	274.65 (173.9, 342.7)	188 (98.35, 405.35)
pH	7.31 (7.235, 7.345)	7.285 (7.245, 7.335)	7.33 (7.21, 7.36)
PaO_2_ (mmHg)	88 (77.5, 109)	90 (77.5, 112.5)	83 (75.5, 106.5)
PaCo_2_ (mmHg)	38 (35.75, 49)	38 (34.5, 40)	48 (37.25, 49.5)
HCO_3_ (mmol/L)	19 (17.75, 20.25)	18 (16.5, 20.5)	19 (18.75, 21.75)
Base excess (mmol/L)	−7.2 (−9.775, −4.225)	−8.05 (−10.05, −4.85)	−6.2 (−8.5, −2.1)
FiO_2_	0.5 (0.438, 0.663)	0.55 (0.45, 0.675)	0.5 (0.37, 0.637)
PaO_2_/FiO_2_	172.31 (128.49, 255.5)	167.265 (130.475, 3.215)	240 (126.42, 288.035)
O_2_ Saturation (%)	96 (95, 98)	96.5 (95.5, 98)	95 (93, 97.25)
Saturation O_2_/FiO_2_	190 (146.207, 221.248)	176.665 (143.185, 13.33)	200 (144.753, 270.358)

Data are presented as median, 25^th^ and 75^th^ percentiles or as frequency (%). The two groups did not differ significantly (p-value > 0.05 Wilcoxon rank-sum test).

The characteristics of the 21 patients at enrolment (T1) are reported in Table 1. Mortality and length of stay, comorbidities, sources of infection, administered therapies and partial SOFA scores are illustrated in Table 2. No significant differences were found between the two groups at admission. However, though not significantly different, in the NR group one patient died within one week; there was also a higher percentage of deaths within 28 days (43% in NR vs. 14% in R) and a longer hospital stay (33 days in NR vs. 15 days in R).

**Table 2:** Mortality and length of stay, comorbities, sources of infection and partial SOFA scores in the two groups (R: responsive; NR: not responsive to therapy).

	All Patients	R	NR
**Mortality**			
7 days mortality [No (%)]	1 (5%)	0 (0%)	1 (14%)
28 days mortality [No (%)]	5 (24%)	2 (14%)	3 (43%)
In hospital mortality [withdrawal of care]	4 [3] (19% [14%])	1 [1] (7%)	3 [2] (43% [23%])
Length of stay in ICU before discharge (days)^¶^	5 (3.25, 9)	4 (3, 6)	10 (5, 10)
Total length of stay in hospital (days)^¶^	21.5 (11, 32.5)	15 (11, 30)	33 (24.75, 42)
**Comorbidities**			
Acute Heart Failure [No (%)]	12 (57%)	7 (50%)	5 (71%)
Acute Myocardial Infarction [No (%)]	0 (0%)	0 (0%)	0 (0%)
Prolonged arrhythmias [No (%)]	4 (19%)	1 (7%)	3 (43%)
Chronic Organ Insufficiency [No (%)]	19 (91%)	13 (93%)	6 (87%)
Arterial Hypertension [No (%)]	9 (43%)	5 (36%)	4 (57%)
Diabetes Mellitus [No (%)]	8 (38%)	6 (43%)	2 (23%)
Coronary arteries diseases [No (%)]	2 (10%)	1 (7%)	1 (14%)
Systolic heart failure [No (%)]	1 (5%)	0 (0%)	1 (14%)
Diastolic heart failure [No (%)]	0 (0%)	0 (0%)	0 (0%)
Cerebrovascular Disease [No (%)]	2 (10%)	2 (14%)	0 (0%)
Peripheral vascular disease [No (%)]	1 (5%)	1 (7%)	0 (0%)
Dementia [No (%)]	2 (10%)	2 (14%)	0 (0%)
Chronic Lung Disease [No (%)]	3 (14%)	1 (7%)	2 (23%)
Rheumatic/connective tissue disease [No (%)]	0 (0%)	0 (0%)	0 (0%)
Inflammatory Bowel Disease [No (%)]	0 (0%)	0 (0%)	0 (0%)
Peptic ulcer [No (%)]	0 (0%)	0 (0%)	0 (0%)
Mild liver disease [No (%)]	0 (0%)	0 (0%)	0 (0%)
Moderate/severe liver Disease [No (%)]	2 (10%)	1 (7%)	1 (14%)
Chronic Kidney Disease [No (%)]	0 (0%)	0 (0%)	0 (0%)
Tumor without metastasis [No (%)]	2 (10%)	1 (7%)	1 (14%)
Hemiplegia/paraplegia [No (%)]	1 (5%)	1 (7%)	0 (0%)
**Source of Infection**			
Respiratory [No (%)]	5 (24%)	3 (21%)	2 (23%)
Abdominal [No (%)]	7 (33%)	3 (21%)	4 (57%)
Urinary Tract [No (%)]	6 (29%)	5 (36%)	1 (14%)
Others [No (%)]	3 (14%)	3 (21%)	0 (0%)
**Therapies**			
Beta-blocker [No (%)]	3 (14%)	2 (14%)	1 (14%)
Ionotropic Drugs [No (%)]	7 (33%)	4 (29%)	3 (43%)
Sedation drugs [No (%)]	21 (100%)	14 (100%)	7 (100%)
Other drugs [No (%)]	19 (91%)	14 (100%)	5 (71%)
Tracheal Intubation [No (%)]	19 (91%)	13 (93%)	6 (87%)
Renal Replacement Therapy [No (%)]	1 (5%)	1 (7%)	0 (0%)
Transfusion [No (%)]	2 (10%)	0 (0%)	2 (23%)
**Partial Sofa Scores**			
Respiratory System	3 (2, 3)	3 (2, 3)	3 (2, 3)
Nervous System	4 (3.75, 4)	4 (4, 4)	4 (3.25, 4)
Cardiovascular System	4 (3.75, 4)	4 (3, 4)	4 (4, 4)
Liver	1 (0, 2)	1 (0, 1)	1 (0.25, 2.75)
Coagulation	0 (0, 1.25)	0 (0, 1)	1 (0, 2.5)
Renal System	1 (0.75, 2)	1 (0, 2)	2 (1, 2.75)

Data are presented as median, 25^th^ and 75^th^ percentiles or as frequency (%). No significant differences were found (p-value > 0.05, Wilcoxon rank-sum test for continuous variables, Fishers’ Exact Test for categorical variables). Despite this, NR had longer ICU stay and higher mortality. ^¶^Indicates that analyses were done on 19 patients (3 patients were excluded as they died in the ICU).

### Metabolic fingerprinting by untargeted metabolomics

**Figure 1:**
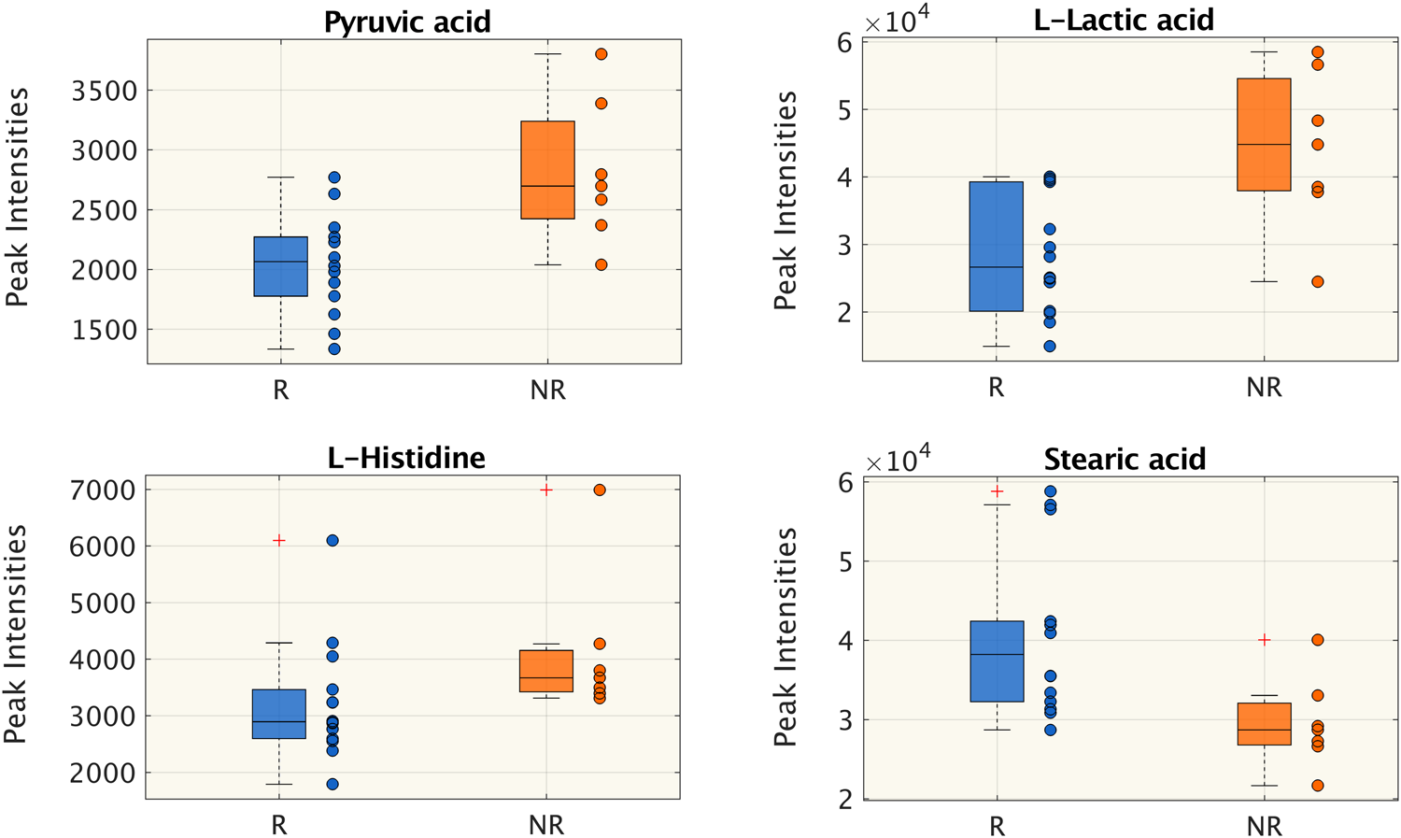
Untargeted metabolomics. Metabolites whose peak intensity is significantly different between responsive (R) and non-responsive (NR) patients at T2 (Wilcoxon rank-sum test p < 0.05, FDR < 0.15). Distributions are shown as box-plots where the central line is the median concentration, the edges of the box are the 25th and 75th percentiles and outliers are defined as 1.5 times the interquartile range and highlighted by +.

**Figure 2:**
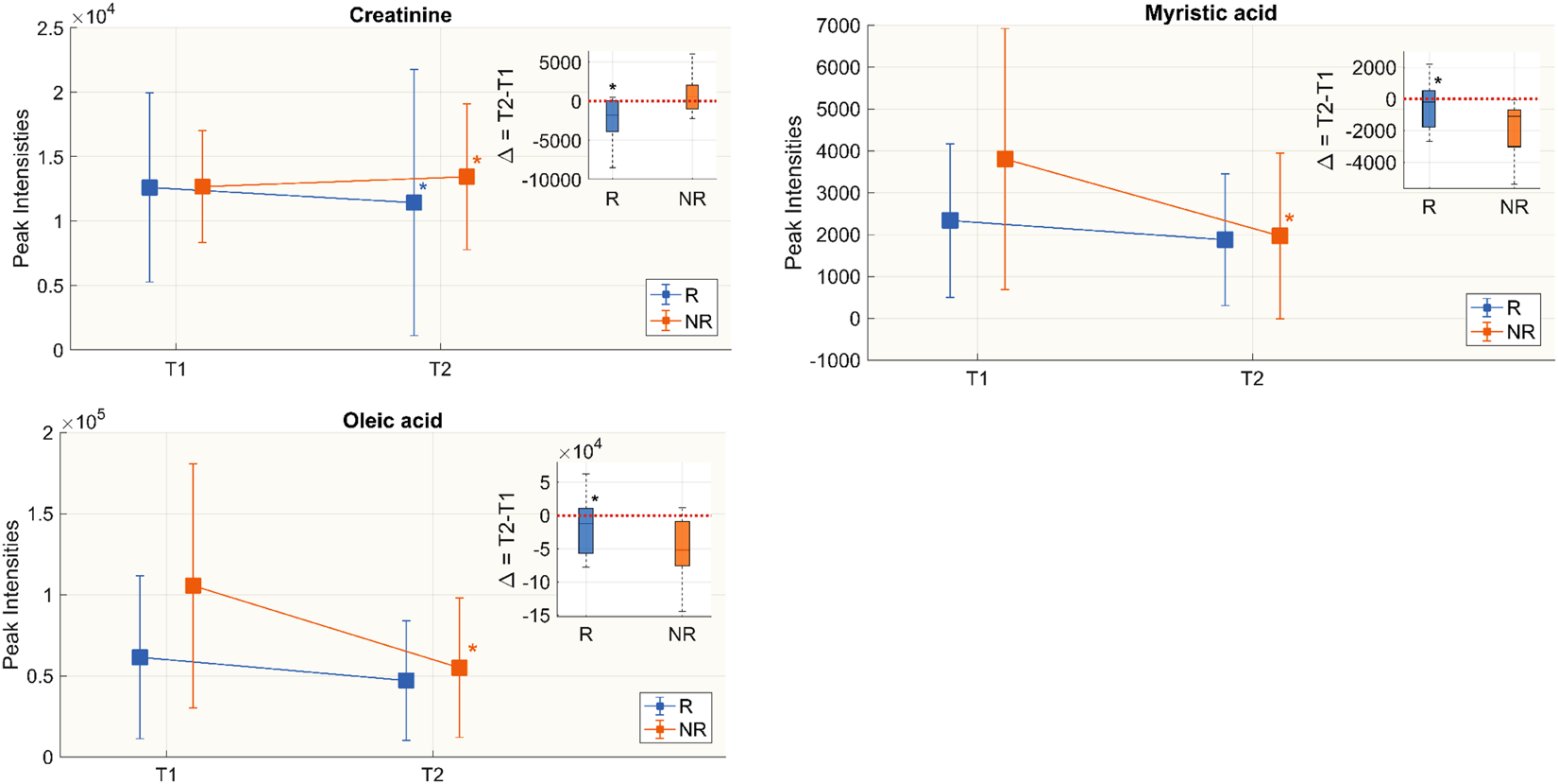
Untargeted metabolomics. Metabolites whose change in peak intensity from T1 to T2 in the two groups is statistically significant. Box-plots in the top right corner show differences in metabolite peak intensity between T1 and T2 expressed as delta (Δ = T2 − T1). We did the Wilcoxon rank-sum test for the delta of the two groups and Wilcoxon signed rank between T1 and T2 in each group separately. Significant differences are marked with *(p-value < 0.05).

A rapid untargeted analysis by flow injection-TOF-MS was done to screen for metabolic features significantly characterizing the responsiveness (R) group and non-responsive (NR) group to therapy in septic shock patients. Statistical analyses on the species identified from the untargeted approach showed that at T1 the two groups were similar and most of the differences were seen at T2. None of the species identified had a significant difference between R and NR at T1, except 4 species: stearic acid was lower in NR, while pyruvic acid, lactic acid and histidine were higher (Fig. 1). The changes in peak intensities from T1 to T2 were verified in the two groups separately and then compared (Table 3). There was a general increase in circulating essential aminoacids such as arginine, tyrosine, threonine and lysine at T2 in R and NR, but only lysine and threonine rose significantly in both groups. Similarly, the abundance of acetylcarnitine was significantly lower in R and NR (Table 3). Only NR showed a significant reduction in circulating fatty acids, mainly saturated and monosaturated (myristic acid, palmitoleic acid, palmitic acid, oleic acid and stearic acid). The endogenous kynuramine, derived from tryptophan, increased with time in both groups, although significantly only in R. The trend (Δ = T2 − T1) showed three species significantly differed between the two groups: creatinine decreased in R and increased in NR, while myristic acid and oleic acid significantly decreased in NR only (Fig. 2).

**Table 3:** Metabolite peak intensities from T1 to T2 in the two groups (untargeted approach).

	Responsive to therapy (R)						Not responsive to therapy (NR)				
	T1	T2		Δ = T2 − T1			T1	T2		Δ = T2 − T1	
Creatinine	12116 (10211, 17348)	11109 (8507, 13271)	*	−1797 (−3691, −377)	↓	§	13371 (10815, 15693)	13749(10223, 19343)		792 (−557, 2907)	↑
L-Arginine	4780 (3369, 5930)	6528 (5183, 7638)		2113 (552, 3812)	↑		3434 (3200, 4627)	7275 (5063, 8561)	*	2958 (1379, 3837)	↑
L-Acetylcarnitine	14079 (7830, 17703)	9163 (6284, 11268)	*	−4143 (−10588, 1024)	↓		22939 (11352, 28847)	14851 (9771, 19232)	*	−4307 (−12256, −2900)	↓
L-Threonine	1155 (819, 1518)	1747 (1415, 2296)	*	816 (−53, 1075)	↑		1038 (823, 1518)	1754 (1520, 2214)	*	481 (238, 1005)	↑
Taurine	1302 (929, 1837)	859 (729, 1264)	*	−282 (−486, −83)	↓		1516 (1032, 1902)	1095 (883, 1421)		−362 (−975, 44)	↑
Kynuramine	1496 (1418, 1688)	1732 (1589, 1776)	*	122 (28, 285)	↑		1381 (1233, 1481)	1584 (1320, 1701)		72 (−2.55, 254)	↑
L-Tyrosine	874 (788, 1040)	1181 (867, 1476)	*	105 (22, 667)	↑		927 (801, 1089)	1167 (931, 1740)		281 (194, 403)	↑
Citric acid	26765 (16429, 33356)	16942 (7661, 24011)	*	−7611 (−12761,−1338)	↓		33185 (24219, 44715)	27909 (22868, 32550)		−8845 (−12989, −1882)	↓
L-Lysine	1133 (891, 1405)	1491 (1280, 2053)	*	336 (90, 1190)	↑		1149 (840, 1626)	1515 (1324, 1973)	*	446 (254, 819)	↑
Stearic acid	37344 (32419, 58541)	38207 (32244, 42399)		−3464 (−9555, 2738)	↑		38710 (31144, 51784)	28711 (26782, 32069)	*	−11046 (−14567, −6515)	↓
Myristic acid	2145 (1463, 3917)	1511 (972, 3111)		−457 (−1804, 284)	↓	§	3217 (1971, 6396)	1024 (816, 2326)	*	−1365 (−3421, −991)	↓
Palmitoleic acid	5130 (2286, 9028)	4338 (1625, 5849)		−1586 (−4475, 1268)	↓		5852 (4761, 11911)	2271 (1798, 4542)	*	−3707 (−8859, −2472)	↓
Palmitic acid	61453 (47464, 76280)	58577 (48918, 61999)		−5683 (−18696, 5115)	↓		68002 (52214, 96968)	47928 (36302, 53361)	*	−21621 (−48579,−11540)	↓
Oleic acid	46791 (31181, 92927)	43499 (27535, 85340)		−11901 (−56910, 11496)	↓	§	94476 (76885, 179484)	42356 (24828, 65253)	*	−63747 (−87753,−33127)	↓

Significant differences between T1 and T2 are marked with *(Wilcoxon signed-rank test p < 0.05), ^§^marks differences in the delta between R and NR (Wilcoxon rank-sum test p < 0.05). The arrows indicate whether the metabolite concentration at T2 is lower (↓) or higher (↑) than at T1.

### Metabolic profiling by targeted metabolomics

We applied a mass spectrometry-based quantitative metabolomics profiling to unambiguously identify and quantify glycerophospholipids, aminoacids, acylcarnitines and biogenic amines in the plasma of the study subjects.

Similarly to the untargeted metabolomics approach, univariate statistical analyses showed that most of the differences in metabolite levels arose at T2.

**Figure 3:**
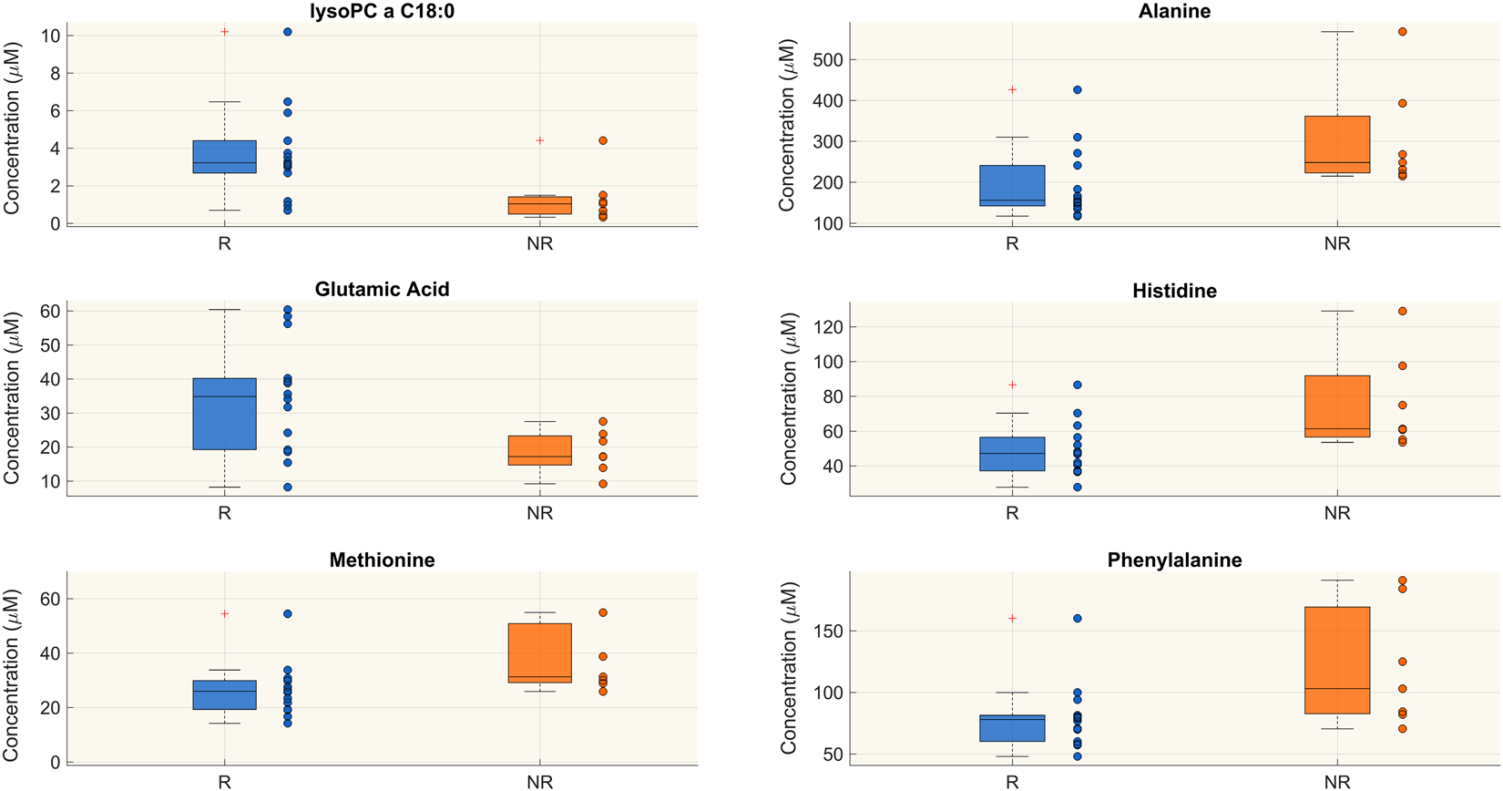
Targeted metabolomics. Metabolites whose concentration (μM) is significantly different between responsive (R) and non-responsive (NR) patients at T2 (Wilcoxon rank-sum test p-value < 0.05, FDR < 0.15). The figure shows only 6 out of 23 metabolites (see Table 4) significantly differing in the two groups at T2 as example. Distributions are shown as box-plots where the central line is the median concentration, the edges of the box are the 25th and 75th percentiles and the outliers are defined as 1.5 times the interquartile range and highlighted by +.

Only four metabolites had significant differences in concentrations in the two groups at T1 (Supplemental Table S1 and Figure S1), and 23 metabolites at T2 (Table 4). At T2 the plasma levels of six species of lysophosphatidylcholines (lysoPCs), seven diacyl-phosphatidylcholines (PCaa), two acyl-alkyl phosphatidylcholines (PCae), two long-chain sphingomyelins (SM) and glutamic acid were lower in NR than in R, whereas there was greater abundance in NR for aminoacids such as alanine, methionine, phenylalanine and histidine (Table 4 and Fig. 3).

**Table 4:** Species identified by the targeted approach which are significantly different in the R and NR groups at T2 (Wilcoxon rank-sum test p < 0.05 and FDR < 0.15).

	R	NR	R vs NR
lysoPC a C16:0	15.700 (10.300, 17.500)	4.020 (1.400, 6.572)	↑
lysoPC a C16:1	0.604 (0.470, 0.690)	0.158 (0.099, 0.238)	↑
lysoPC a C18:0	3.220 (2.680, 4.400)	1.040 (0.494, 1.415)	↑
lysoPC a C18:1	6.090 (3.010, 6.770)	1.470 (1.170, 2.200)	↑
lysoPC a C18:2	6.075 (2.330, 8.060)	1.160 (0.916, 2.310)	↑
lysoPC a C20:3	0.474 (0.333, 0.834)	0.222 (0.147, 0.275)	↑
PC aa C36:0	2.645 (1.900, 3.270)	1.880 (1.580, 1.980)	↑
PC aa C36:3	188 (136, 227)	120 (110.25, 133.5)	↑
PC aa C36:6	0.888 (0.841, 1.050)	0.570 (0.534, 0.717)	↑
PC aa C38:3	36.750 (29.200, 47.700)	25.300 (18.750, 30.025)	↑
PC aa C38:6	88.150 (70.800, 101.000)	62.200 (57.175, 77.900)	↑
PC aa C40:5	6.670 (6.220, 7.350)	5.500 (4.575, 6.715)	↑
PC aa C40:6	23.800 (17.700, 25.400)	19.400 (16.025, 19.800)	↑
PC aa C42:2	0.154 (0.121, 0.176)	0.111 (0.102, 0.116)	↑
PC ae C38:0	1.415 (1.280, 1.620)	0.970 (0.954, 1.238)	↑
PC ae C38:3	4.040 (2.960, 4.480)	2.920 (2.353, 3.075)	↑
SM C18:0	7.385 (6.880, 8.770)	6.430 (4.728, 6.750)	↑
SM C24:0	1.235 (1.020, 1.560)	0.954 (0.882, 1.115)	↑
Alanine	156 (142, 241)	248 (222.75, 361.75)	↓
Glutamic acid	34.850 (19.200, 40.200)	17.200 (14.700, 23.275)	↑
Histidine	47.100 (37.100, 56.400)	61.300 (56.500, 91.850)	↓
Methionine	25.950 (19.300, 29.900)	31.300 (29.175, 50.875)	↓
Phenylalanine	77.900 (60.100, 81.400)	103 (82.650, 169.250)	↓

Concentrations are presented as median, 25^th^ and 75^th^ percentiles. The arrows indicate whether the metabolite concentration in R is lower (↓) or higher (↑) than NR.

No metabolites changed significantly in the NR group from T1 to T2, whereas 54 significantly changed in R. In the R group eight species of lysoPCs, seventeen PCaa, twenty-two PCae, eleven SM, seven amino acids (AAs) and two biogenic amines significantly increased from T1 to T2, while histidine, creatinine and taurine decreased significantly. For 38 metabolites the trends (Δ = T2 − T1) were different between R and NR (Table 5).

**Table 5:** Metabolite concentrations (µM) from T1 to T2 in the two groups.

	Responsive to therapy (R)						Not responsive to therapy (NR)			
	T1	T2		Δ = T2 − T1	Trend		T1	T2	Δ = T2 − T1	Trend
lysoPC a C16:0	3.825 (2.320, 5.910)	15.7 (10.3, 17.5)	*	10.205 (2.77, 13.66)	↑	§	2.780 (1.233, 4.902)	4.020 (1.400, 6.572)	0.250 (0.146, 1.648)	↑
lysoPC a C16:1	0.185 (0.124, 0.254)	0.604 (0.470, 0.690)	*	0.383 (0.118, 0.525)	↑	§	0.116 (0.090, 0.160)	0.158 (0.099, 0.238)	0.012 (−0.002, 0.107)	↑
lysoPC a C17:0	0.139 (0.102, 0.191)	0.312 (0.124, 0.376)	*	0.131 (0.025, 0.199)	↑		0.094 (0.059, 0.145)	0.110 (0.080, 0.165)	0.005 (−0.012, 0.064)	↑
lysoPC a C18:0	0.902 (0.603, 1.650)	3.220 (2.680, 4.400)	*	2.054 (0.762, 3.330)	↑	§	0.683 (0.382, 1.252)	1.040 (0.494, 1.415)	0.161 (0.071, 0.403)	↑
lysoPC a C18:1	1.170 (1.070, 1.970)	6.090 (3.010, 6.770)	*	4.160 (1.070, 5.320)	↑	§	1.020 (0.709, 1.360)	1.470 (1.170, 2.200)	0.513 (0.242, 0.964)	↑
lysoPC a C18:2	1.215 (1.050, 1.380)	6.075 (2.330, 8.060)	*	3.455 (1.123, 6.732)	↑	§	0.758 (0.572, 0.990)	1.160 (0.916, 2.310)	0.697 (0.212, 1.355)	↑
lysoPC a C20:3	0.222 (0.145, 0.333)	0.474 (0.333, 0.834)		0.245 (0.104, 0.600)	↑	§	0.165 (0.116, 0.229)	0.222 (0.147, 0.275)	0.045 (0.022, 0.056)	↑
lysoPC a C20:4	0.485 (0.359, 0.823)	1.430 (0.800, 1.830)		0.821 (0.262, 1.241)	↑	§	0.478 (0.367, 0.688)	0.456 (0.375, 0.985)	0.059 (−0.006, 0.314)	↑
PC aa C28:1	1.480 (1.280, 1.900)	1.805 (1.460, 2.120)	*	0.310 (−0.070, 0.520)	↑		1.40 (1.105, 1.770)	1.25 (1.083, 2.313)	0.078 (−0.265, 0.430)	↑
PC aa C32:3	0.948 (0.828, 1.130)	1.110 (0.949, 1.420)	*	0.152 (0.066, 0.270)	↑		1.16 (0.816, 1.258)	0.89 (0.850, 1.249)	0.087 (0.349, 0.147)	↑
PC aa C34:3	22.95 (17.9, 33.9)	38.85 (24.9, 47.4)	*	10.00 (0.700, 18.550)	↑		23.50 (14.20, 29.00)	23.70 (14.85, 34.25)	1.300 (−1.725, 3.325)	↑
PC aa C34:4	1.58 (1.29, 1.86)	2.21 (1.38, 2.48)	*	0.560 (0.190, 0.920)	↑		1.48 (1.295, 1.693)	1.160 (1.143, 1.600)	−0.141(−0.255, −0.023)	↓
PC aa C36:1	55.8 (45.5, 70.9)	81.75 (58.6, 95.4)	*	20.15 (−0.500, 41.300)	↑		50.90 (44.20, 55.40)	63.60 (48.20, 82.25)	6.500(1.250, 21.850)	↑
PC aa C36:2	264.5 (182, 325)	361.5 (324, 495)	*	121 (9, 221)	↑		222 (207, 223.75)	298 (229.5, 313.25)	74 (3.25, 102.25)	↑
PC aa C36:3	124.5 (106, 147)	188 (136, 227)	*	76 (2, 97.6)	↑		104 (88.60, 121)	120 (110.25, 133.5)	18.0 (−7.25, 19.80)	↑
PC aa C38:0	1.630 (1.250, 2.080)	1.745 (1.380, 2.730)	*	0.340 (−0.140, 0.802)	↑	§	2.010 (1.978, 2.237)	1.670 (1.585, 1.778)	−0.440 (−0.548,−0.245)	↓
PC aa C38:1	0.601 (0.353, 0.833)	0.661 (0.408, 0.740)		0.079(−0.021, 0.259)	↑	§	0.602 (0.385, 1.107)	0.417 (0.347, 0.532)	−0.097 (−0.938, 0.003)	↓
PC aa C38:3	28.8 (23.4, 41.2)	36.750 (29.200, 47.700)	*	9.25 (−1.000, 11.900)	↑	§	28.30 (16.60, 31.175)	25.30 (18.75, 30.025)	−0.90 (−6.300, 2.200)	↓
PC aa C38:5	37.8 (29.4, 49.2)	47.85 (44.2, 51.9)	*	13.85 (2.9, 19.4)	↑	§	49.6 (41.225, 52.9)	38.7 (30.7, 47.025)	−7.100 (−13.450, 2.175)	↓
PC aa C38:6	78.45 (51.6, 90.4)	88.15 (70.8, 101)	*	15.7 (5.9, 33.5)	↑	§	69.8 (57.5, 91.2)	62.2 (57.175, 77.9)	−11.30 (−17.85, −0.825)	↓
PC aa C40:4	1.655 (1.410, 2.020)	2.115 (1.750, 2.630)		0.39 (−0.13, 0.860)	↑	§	1.840 (1.250, 2.473)	1.590 (1.478, 2.003)	−0.310 (−0.540, 0.253)	↓
PC aa C40:5	5.760 (3.700, 7.180)	6.670 (6.220, 7.350)	*	1.515 (−0.26, 3.08)	↑	§	5.900 (4.890, 7.527)	5.500 (4.575, 6.715)	−0.680 (−1.485, 0.512)	↓
PC aa C40:6	19.45 (15.2, 20.4)	23.8 (17.7, 25.4)	*	5.450 (2.500, 10.600)	↑	§	19.4 (14.25, 23.725)	19.4 (16.025, 19.8)	0.000 (−3.775, 0.900)	//
PC aa C42:2	0.122 (0.101, 0.150)	0.154 (0.121, 0.176)	*	0.037 (−0.017, 0.067)	↑		0.110 (0.089, 0.158)	0.111 (0.102, 0.116)	−0.008 (−0.039, 0.015)	↓
PC aa C42:4	0.085 (0.065, 0.112)	0.105 (0.077, 0.151)	*	0.012 (0.002, 0.037)	↑		0.086 (0.080, 0.127)	0.079 (0.075, 0.110)	−0.004 (−0.042, 0.018)	↓
PC ae C34:2	13.4 (11, 19.1)	15.3 (11.1, 23.8)	*	2.80 (0.000, 6.230)	↑		14.70 (11.650, 16.200)	11.80 (11.025, 16.525)	−0.300 (−2.625, 1.212)	↓
PC ae C34:3	6.205 (4.340, 7.470)	6.575 (4.900, 9.800)		0.070 (−0.600, 2.500)	↑	§	6.760 (4.633, 8.373)	4.490 (3.763, 6.342)	−1.570 (−3.197,−0.070)	↓
PC ae C36:2	16.8 (14.6, 20.9)	21.9 (15.2, 27.6)	*	4.150 (0.400, 9.700)	↑		18.20 (15.425, 21.150)	17.20 (15.550, 24.925)	2.60 (−1.825, 3.210)	↑
PC ae C36:3	6.580 (5.270, 8.760)	7.065 (6.210, 12.200)	*	1.140 (−0.260, 2.800)	↑	§	6.490 (6.460, 7.298)	5.680 (5.215, 6.982)	−0.930 (−1.225, 0.212)	↓
PC ae C36:4	15.7 (13.4, 18.2)	16.85 (13.2, 18.7)		0.950 (−0.600, 3.650)	↑	§	15.7 (14.675, 18.3)	13.2 (12.225, 14.1)	−2.500 (−5.675,−1.700)	↓
PC ae C36:5	9.88 (8.99, 11.9)	11.1 (9.99, 12.2)		0.920 (−1.200, 3.210)	↑	§	10.2 (8.402, 14.125)	8.470 (7.172, 10.372)	−3.700 (−3.850,−0.845)	↓
PC ae C38:0	1.265 (1.120, 1.350)	1.415 (1.280, 1.620)	*	0.185 (0.100, 0.590)	↑	§	1.190 (1.080, 1.395)	0.970 (0.954, 1.238)	−0.110 (−0.199,−0.084)	↓
PC ae C38:1	0.407 (0.276, 0.614)	0.649 (0.518, 1.020)	*	0.162 (0.075, 0.387)	↑		0.596 (0.248, 0.725)	0.690 (0.582, 0.870)	0.158 (−0.011, 0.250)	↑
PC ae C38:2	1.725 (1.530, 2.040)	1.845 (1.400, 2.730)	*	0.360 (−0.040, 0.746)	↑		1.860 (1.763, 2.013)	1.910 (1.845, 2.113)	−0.030 (−0.088, 0.335)	↓
PC ae C38:3	3.340 (2.760, 3.960)	4.040 (2.960, 4.480)		0.800 (−0.570, 1.290)	↑	§	3.160 (2.530, 4.058)	2.920 (2.353, 3.075)	−0.590 (−1.000,−0.198)	↓
PC ae C38:4	11.75 (7.42, 14.2)	10.71 (9.1, 14.1)		0.350 (−0.800, 2.400)	↑	§	14.1 (11.6, 15.15)	10 (8.668, 12.1)	−2.540 (−4.033,−1.975)	↓
PC ae C38:5	12.9 (11.4, 14.4)	13.3 (10.2, 17.4)		1.750 (−1.000, 4.400)	↑	§	16.2 (13.9, 18.875)	12.9 (10.725, 13.8)	−4.100 (−5.880,−3.000)	↓
PC ae C38:6	4.910 (4.050, 5.720)	4.720 (4.280, 7.240)		0.685 (−0.100, 1.390)	↑	§	5.270 (4.880, 6.490)	3.940 (3.760, 4.133)	−1.760 (−2.057,−1.080)	↓
PC ae C40:2	1.010 (0.851, 1.140)	1.215 (1.060, 1.460)	*	0.219 (0.120, 0.310)	↑	§	1.180 (1.000, 1.335)	1.010 (0.933, 1.275)	−0.120 (−0.377,−0.067)	↓
PC ae C40:5	2.040 (1.770, 2.370)	2.405 (1.600, 2.700)		0.390 (−0.100, 0.860)	↑	§	2.660 (2.395, 2.767)	2.110 (1.822, 2.190)	−0.700 (−0.935,−0.298)	↓
PC ae C40:6	2.750 (2.390, 3.550)	2.840 (2.640, 3.910)	*	0.565 (0.220, 0.840)	↑	§	3.020 (2.740, 3.775)	2.570 (2.252, 2.865)	−0.410 (−1.168,−0.338)	↓
PC ae C42:2	0.223 (0.188, 0.259)	0.250 (0.222, 0.294)	*	0.219 (0.120, 0.310)	↑		0.258 (0.195, 0.313)	0.265 (0.209, 0.295)	−0.036 (−0.058, 0.041)	↓
PC ae C42:3	0.339 (0.243, 0.395)	0.395 (0.256, 0.512)	*	0.078 (0.004, 0.140)	↑	§	0.286 (0.266, 0.349)	0.331 (0.261, 0.362)	0.002 (−0.053, 0.040)	//
PC ae C42:4	0.272 (0.217, 0.415)	0.300 (0.242, 0.482)		0.036 (0.009, 0.090)	↑	§	0.316 (0.272, 0.410)	0.251 (0.216, 0.297)	−0.060 (−0.134,−0.005)	↓
PC ae C42:5	0.875 (0.693, 0.944)	0.984 (0.579, 1.160)		0.066 (−0.146, 0.182)	↑	§	1.020 (0.956, 1.293)	0.887 (0.791, 1.051)	−0.169 (−0.365,−0.074)	↓
PC ae C44:5	0.421 (0.302, 0.464)	0.430 (0.231, 0.535)		−0.009 (−0.075, 0.154)	↓	§	0.487 (0.438, 0.639)	0.376 (0.323, 0.492)	−0.111 (−0.149,−0.090)	↓
PC ae C44:6	0.319 (0.234, 0.345)	0.342 (0.247, 0.435)		0.053 (−0.015, 0.105)	↑	§	0.405 (0.354, 0.517)	0.304 (0.259, 0.361)	−0.126 (−0.177,−0.031)	↓
SM (OH) C14:1	2.295 (1.830, 3.050)	3.065 (2.220, 3.380)	*	0.425(0.160, 0.890)	↑		2.660 (2.023, 2.830)	2.210 (2.152, 3.325)	0.000 (−0.642, 0.352)	//
SM (OH) C16:1	0.948 (0.761, 1.260)	1.300 (1.010, 1.390)		0.250 (0.041, 0.416)	↑		1.170 (0.998, 1.405)	1.030 (0.849, 1.293)	−0.240 (−0.353, 0.054)	↓
SM (OH) C22:1	0.731 (0.684, 0.915)	0.837 (0.727, 1.140)	*	0.082(−0.004, 0.289)	↑		0.738 (0.574, 1.065)	0.732 (0.516, 0.916)	−0.130 (−0.258, 0.146)	↓
SM (OH) C24:1	0.071 (0.053, 0.098)	0.077 (0.052, 0.098)		0.003 (−0.011, 0.023)	↑	§	0.065 (0.054, 0.095)	0.054 (0.041, 0.069)	−0.015 (−0.033,−0.010)	↓
SM C16:0	35.95 (31, 39.9)	48.95 (38.7, 54.9)	*	13.3 (3.8, 18.3)	↑	§	42.9 (30.475, 48.575)	40.6 (37.9, 45.275)	−0.900 (−6.175, 6.100)	↓
SM C16:1	5.570 (5.100, 6.520)	6.815 (5.710, 8.650)	*	1.260 (0.220, 3.190)	↑	§	6.610 (5.390, 7.965)	6.410 (5.200, 7.570)	0.060 (−1.385, 0.643)	↑
SM C18:0	6.245 (5.380, 7.030)	7.385 (6.880, 8.770)	*	1.300 (0.450, 2.620)	↑	§	6.010 (5.343, 9.427)	6.430 (4.728, 6.750)	−1.580 (−3.072, 0.520)	↓
SM C18:1	3.020 (2.890, 3.700)	3.585 (2.950, 4.210)	*	0.525 (0.200, 0.840)	↑	§	3.560 (2.887, 4.277)	2.960 (2.325, 3.502)	−0.880 (−1.333, 0.065)	//
SM C20:2	0.127 (0.108, 0.142)	0.169 (0.106, 0.258)	*	0.045 (0.005, 0.116)	↑		0.168 (0.128, 0.182)	0.117 (0.107, 0.199)	0.008 (−0.041, 0.044)	↑
SM C24:0	1.050 (0.838, 1.380)	1.235 (1.020, 1.560)	*	0.158 (0.050, 0.465)	↑		1.000 (0.747, 1.370)	0.954 (0.882, 1.115)	−0.056 (−0.247, 0.145)	↓
SM C24:1	3.410 (2.740, 4.310)	4.885 (3.580, 5.310)	*	0.565 (0.050, 2.420)	↑		4.060 (2.388, 5.902)	3.680 (3.293, 4.188)	−0.580 (−1.877, 0.998)	↓
Arginine	42.25 (29.2, 58.2)	64 (56.1, 86)	*	23.15 (3.500, 49.20)	↑		40.70 (31.750, 59.850)	69.20 (51.125, 86.200)	25.10 (10.425, 54.075)	↑
Histidine	64.2 (51, 72.7)	47.1 (37.1, 56.4)	*	−15.55 (−24.3, −2.4)	↓		64.70 (51.525, 80.650)	61.30 (56.500, 91.850)	1.00 (−3.750, 7.375)	↑
Lysine	114.5 (103, 141)	161 (135, 241)	*	49.95 (6.0, 130)	↑		146 (93.925, 271.750)	190 (155.250, 217.750)	55 (38.825, 81.575)	↑
Ornitine	23.65 (20.1, 31.1)	58.95 (31.2, 84.4)	*	12.60 (−2.70, 65.10)	↑		31.30 (22.625, 42.875)	69.40 (47.675, 92.650)	34.00 (15.250, 76.493)	↑
Serine	41.05 (33.5, 50.3)	63.1 (55.2, 84.4)	*	22.30 (5.10, 29.80)	↑		43.60 (38.275, 56.375)	66.60 (54.800, 81.300)	26.30 (7.900, 48.300)	↑
Threonine	56.2 (37.4, 68.7)	78.35 (60, 89.6)	*	24.70 (−3.80, 43.20)	↑		53.70 (42.550, 74.100)	80.40 (72.200, 102.300)	19.80 (15.625, 35.325)	↑
Trptophan	16.25 (11.9, 21.6)	27.6 (20.5, 35.1)	*	9.335 (0.200, 19.10)	↑		13.50 (9.720, 30.275)	22.20 (21.875, 70.950)	14.88 (4.050, 28.010)	↑
Tyrosine	40.2 (32.9, 45.9)	50.95 (37.4, 62.4)	*	9.05 (−4.900, 31.100)	↑		52.00 (36.675, 62.500)	48.10 (37.050, 90.875)	2.80 (−2.475, 28.450)	↑
Creatinine	91.95 (64.7, 134)	74 (55.9, 98.1)	*	−14.65 (−35.90, −7.0)	↓		92.40 (82.475, 132.250)	106 (77.225, 168.500)	1.50 (−9.450, 19.60)	↑
Met SO	0.552 (0.001, 0.735)	0.800 (0.591, 1.190)	*	0.220 (−0.062, 0.859)	↑		0.905 (0.116, 1.423)	1.380 (0.227, 2.768)	0.000 (−0.075, 2.196)	↓
Taurine	27.15 (14.6, 36.7)	15.7 (10.7, 20.3)	*	−10.55 (−13.40, −4.0)	↑		38.60 (30.125, 40.550)	23.10 (13.900, 29.725)	−14.20 (−25.950, −7.400)	↓
Kynurenine	3.610 (2.530, 4.850)	3.325 (2.630, 6.450)		0.215 (−0.630, 1.780)	↑	§	4.110 (2.848, 4.633)	6.010 (4.840, 9.938)	1.520 (1.380, 5.632)	↑

Significant differences between T1 and T2 are marked with *(Wilcoxon sign-rank test p < 0.05), ^§^marks differences in the delta between R and NR (Wilcoxon rank-sum test p < 0.05). Metabolite abundance changed significantly from T1 to T2 in the NR group.

**Figure 4:**
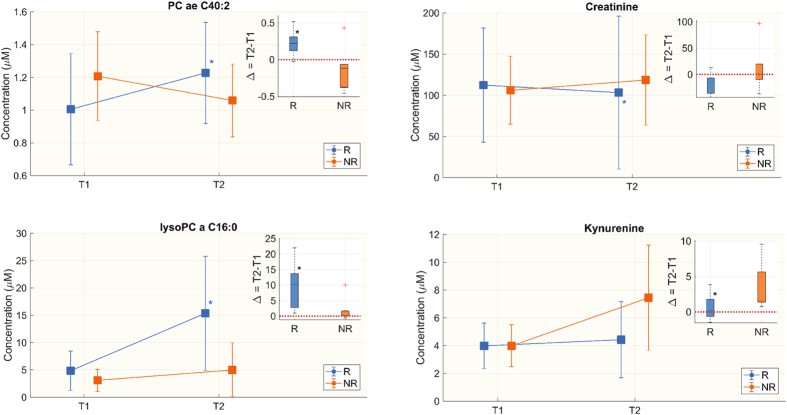
Targeted metabolomics. Metabolites whose concentration (μM) from T1 to T2 in the two groups is statistically different. The figure shows only 4 metabolites as an example of those differing overtime (see Table 5). Box-plots in the top right corner show the differences in metabolite concentrations between T1 and T2, expressed as delta (Δ = T2 − T1). We did the Wilcoxon rank-sum test for the delta of the two groups and Wilcoxon signed rank between T1 and T2 in each group separately. Significant differences are marked with *(p-value < 0.05).

Moreover, kynurenine, a product of the trypthophan catabolism increased in the NR group significantly more than R group as shown in Fig. 4.

### Regression analysis for targeted metabolomics data

As combinations of features can give more information than features considered singly, we used classification models with the aim of identifying a set of metabolites that are mostly associated with the target class, i.e. patients not responsive to therapy (NR). The coefficients of the models obtained are reported in Supplemental Table S2. The interpretation of the coefficients in the logistic regression it is not trivial. If we express the odd-ratio as exponential of linear combination of the independent variables, we can say that if the coefficient *β*_*i*_ is positive then the increase of the feature *x*_*i*_ will be associated with the increase of the odd ratio, i.e. the probability to belong to class 1 (NR) is higher than to class 0 (R), given all other *x*_*j*_ variables being equal. On the contrary, if the coefficient *β*_*i*_ is negative then the increase of the feature *x*_*i*_ will be associated with the decrease of the odd ratio, i.e. the probability to belong to class 1 is lower than to class 0.

**Figure 5:**
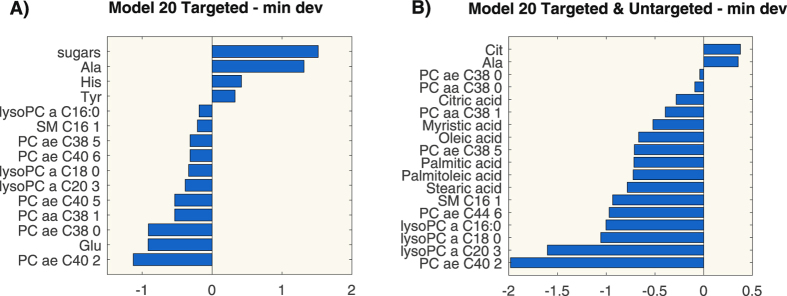
Coefficients values of the logistic regression models for targeted metabolomics (panel A) and for integration of targeted and untargeted metabolomics (panel B).

Three metabolites were selected in all models: PC ae C40:2, PC ae C38:0 and alanine. Figure 5A shows the coefficient values of the model built according to the criterion of minimal deviance on the first 20 ranked features. All the obtained models correctly classify the observations in the testing set so the performances are better than the ones from the metabolites considered individually for patient classification (the average AUC of the metabolites is below 0.8, as shown in Supplemental Figure S2).

### Regression models for targeted and untargeted metabolomics data

We built the integrated models by using 10 features from untargeted metabolomics data and the first 20 ranked metabolites from targeted analysis, as explained in the Methods section.

We can notice that the set of features selected includes again lysoPCs, PCs and alanine. Moreover, six further species not measured by targeted analysis entered in the models: stearic acid, palmitoleic acid, palmitic acid, oleic acid, myristic acid and citric acid. The coefficients of the models are reported in Supplemental Table S3. Figure 5B shows the coefficients of the model built according to the criterion of minimal deviance on the first 20 ranked features. We can notice that alanine, PC ae C38:0, PC aa C38:1, myristic acid and palmitoleic acid are selected by all models. It is worth to underline that PC ae C38:0 and alanine have coefficients with the same sign as the coefficients of the models built on targeted metabolomics data only.

### Discriminant Analysis

The coefficient values of the LDA models and the VIP scores of the PLS-DA models built on the first 10 and 20 ranked features after mRMR are reported in Tables 6 and 7 for targeted metabolomics and for targeted plus untargeted metabolomics data respectively. We cannot use the entire subset of 30 features due to the lower number of observations (i.e. 21 patients only). In fact, the computation of the boundary region requires the covariance matrix would be invertible and this is not the case.

**Table 6:** VIP scores of PLS-DA and coefficients of LDA for the targeted metabolomics models.

Metabolites	PLS DA 20	VIP PLS DA 10	COEF LDA
PC ae C38:0	1.351	—	—
PC ae C38:5	1.275	—	—
PC ae C44:6	1.210	—	—
PC ae C40:2	1.209	1.263	−3.535
PC ae C40:6	1.207	1.241	2.699
PC ae C40:5	1.176	—	—
PC aa C38:0	1.171	1.185	−4.278
PC ae C38:4	1.090	—	—
Ala	1.049	1.069	1.978
His	0.939	—	—
PC aa C38:6	0.918	0.972	−0.448
PC aa C38:1	0.875	0.941	−0.612
lysoPC a C16:0	0.851	—	—
lysoPC a C20:3	0.850	0.830	−2.231
lysoPC a C18:0	0.823	0.906	1.343
Cit	0.816	—	—
SM C16:1	0.812	0.888	0.850
Glu	0.782	0.431	—
Tyr	0.579	—	−1.058
sugars	0.487	—	—

**Table 7:** VIP scores of PLS-DA and coefficients of LDA for the integrated targeted and untargeted metabolomics models.

Metabolites	VIP PLS DA 20	VIP PLS DA 10	COEF LDA
PC ae C40:2	1.431	1.271	−2.002
PC ae C40:6	1.418	—	—
PC aa C38:0	1.383	1.244	−3.779
Palmitoleic acid	1.168	0.987	−0.909
Myristic acid	1.162	1.053	2.412
PC aa C38:6	1.144	—	—
Ala	1.134	0.874	1.327
PC aa C38:1	1.120	0.964	−0.671
Oleic acid	1.103	—	—
Palmitic acid	1.088	0.926	−3.425
lysoPC a C18:0	1.058	0.903	2.225
SM C16:1	0.995	0.871	0.285
lysoPC a C20:3	0.960	0.791	−4.209
Stearic acid	0.775	—	—
Citric acid	0.667	—	—
L-Acetylcarnitine	0.631	—	—
Tyr	0.479	—	—
Pyruvic acid	0.362	—	—
Kynuramine	0.343	—	—
L-Lactic acid	0.334	—	—

**Figure 6:**
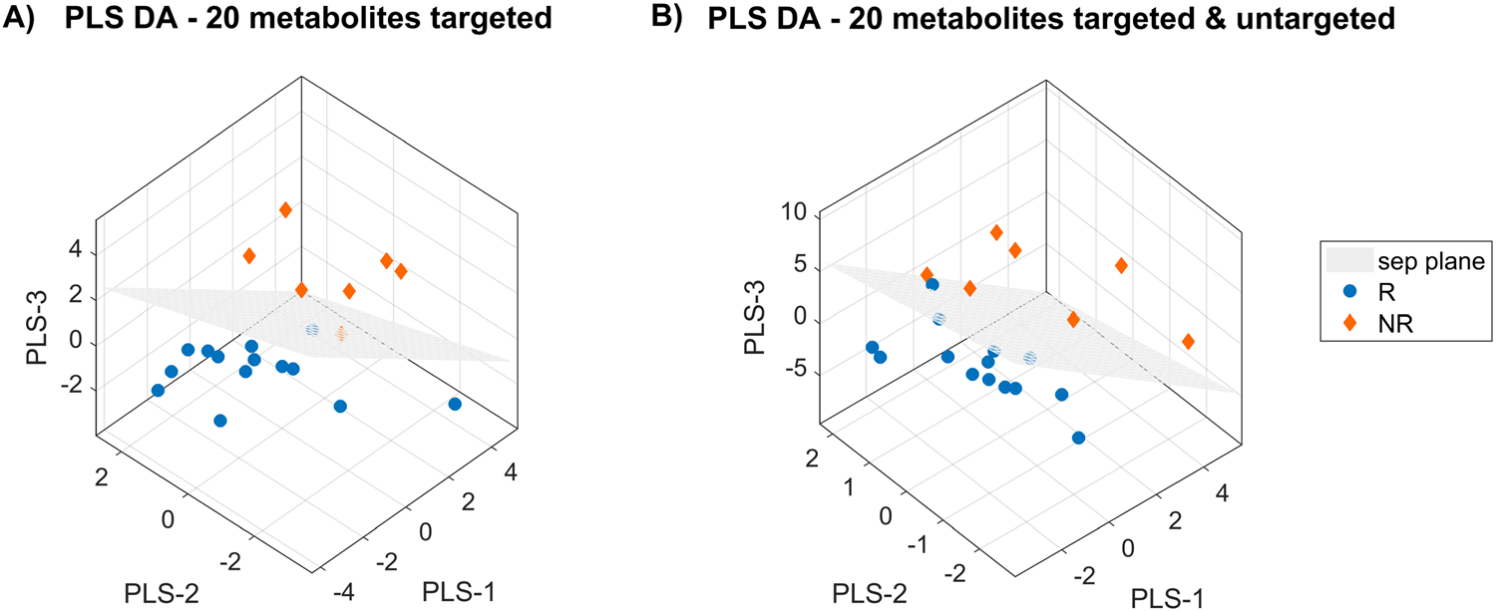
Three-dimensional PLS-DA score plots on 20 features for targeted metabolomics model (panel A) and for targeted and untargeted metabolomics model (panel B).

In the targeted metabolomics model, it is worth to underline that PC ae C38:0, which already played an important role in the regression models, occupies the first position in the VIP ranking when considering 20 features. Similarly, when considering PC ae C40:2, we can notice that it is in the first position when considering 10 features. This latter metabolite also has the highest score in the integrated model. Three-dimensional PLS-DA score plots on 20 features for the two models are shown in Fig. 6 (targeted metabolomics only in panel A; integrated model of targeted and untargeted metabolomics in panel B). In both cases, only one subject (i.e. NR in the targeted model and R in the integrated one) was misclassified by the models.

### Plasma level of sPLA2-IIA

**Figure 7:**
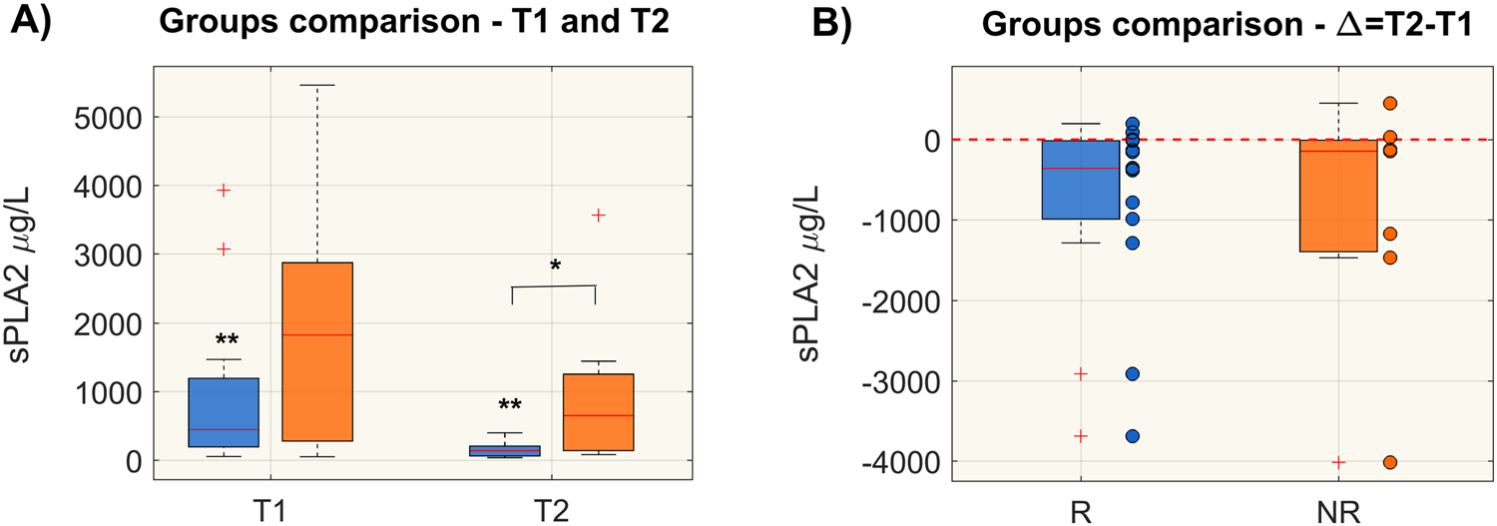
Plasma sPLA2-IIA levels (μg/L) in responsive (R) and non-responsive (NR) patients at T1 and T2 (panel A) and comparison of time trend variation, expressed as delta (Δ = T2 − T1), between the two groups (panel B). Distributions are shown as box-plots where the central line is the median concentration, the edges of the box are the 25th and 75th percentiles and the outliers are defined as 1.5 times the interquartile range and highlighted by +. Significant differences between groups are marked with *(Wilcoxon rank-sum test p-value < 0.05), whereas significant differences from T1 to T2 are marked with **(Wilcoxon sign-rank test p-value < 0.05).

As shown in Fig. 7A, sPLA2-IIA plasma levels were significantly higher in NR than in R patients at T2. To note that responders showed a markedly significant reduction of sPLA2-IIA from T1 to T2. We also compared the sPLA2-IIA variation overtime, expressed as Δ = T2 − T1 (panel B) and sPLA2 –IIA decreased significantly in R only. Some NR patients reduced the level of sPLA2-IIA, however, the population trend was not significant and clear as in R.

## Discussion

Improvement of organ function as assessed by a drop in SOFA score in the first days of sepsis and septic shock is associated with better outcomes^19, 20^, but the mechanisms behind organ improvement remain to be fully elucidated. We made a comprehensive metabolomics study of septic shock patients stratified as responders and not responders to standard therapy on the basis of the changes in SOFA score within the first 48 hours after the enrollment.

To the best of our knowledge, this is the first study to show that plasma metabolome changes are associated with initial responsiveness to therapy in septic shock patients.

Combining untargeted and targeted metabolomics methods by collecting data for fast untargeted MS data acquisition and high-resolution MRM transitions for targeting multiple metabolites, we obtained a wider picture of patients’ metabolic states and their metabolic trajectory during the first 48 hours after ICU admission. Univariate analysis and the classification models confirmed that NR group presented an overall lipidome alteration, as previously reported^14, 15, 18, 21–23^.

Here we showed that in NR patients specific lysophosphatidylcholines species (lysoPC C16:0, C16:1, C18:0, C18:1, C18:2, C20:3) did not significantly change from T1 and T2, whereas in R patients they significantly increased and were markedly higher than in NR at T2 (see Fig. 4 and Table 5). In addition, their respective free fatty acids such as palmitic (C16:0), palmitoleic (C16:1), stearic (C18:0) and oleic (C18:1) acids were significantly lower at T2 in NR only (Table 3).

LysoPCs have a very complex role in metabolism. They are primarily generated by phospholipase A2 (PLA2) enzyme activity, and like this enzyme, they have a direct role in toxic inflammatory responses. Low plasma lysoPC levels have been noted in sepsis patients and systemic treatment with lysoPCs has proved to be therapeutic in rodent models of sepsis and ischemia^24^.

These observations suggest that elevation of plasma levels of these lipids can actually help relieve serious inflammatory conditions. Cunningham *et al*.^24^ reported that specific lysoPCs act as uncompetitive product inhibitors of plasma secreted PLA2 enzymes (sPLA2s), especially under conditions of elevated enzyme activity, thus providing a feedback mechanism for the anti-inflammatory effects of these compounds^25^. Indeed, in the present work we showed that the level of sPLA2-IIA significantly decreased together with an increase of lysoPC species (i.e. lysoPC C16:00 and C18:0) in R patients only. This result is in line with other experimental evidence that reduction of sPLA2-IIA may slow down the inflammatory cascade and increase the probability of responsiveness to therapy^26, 27^ (see also Figure S5).

The reduction in circulating lysoPC in NR patients may simply reflect their enhanced conversion to lysophosphatidic acid (LPA), which induces a multitude of cellular responses through its action on immunologically relevant cells^28^. Conceivably the low lysoPC may also promote an excessive immune response, with detrimental effect in NR patients^21, 29^. Low circulating levels of lysoPC 16 and 18 species have also been reported in inflammatory liver disease^30–32^. NR patients have also showed a marked decrease in PC species, which originate in the liver. The imbalance of lysoPC/PCs cycling suggests that hepatic homeostasis and function is compromised even before any clinical manifestation, and bilirubin alone cannot give a clear picture of the liver’s condition.

In addition, NR patients had lower levels of PC species containing long-chain polyunsaturated fatty acids (LCPUFAs), such as PC aa C38:6, PC aa C36:6, PC aa C40:5, with further elongation/desaturation products. This profile agrees with our previous finding of the different composition of PC species as potential metabolic determinants of mortality in septic shock^14^. Here again we can speculate that, since LCPUFAs reduce T-cell activation and dampen inflammation^33^, a decrease in PC-containing LCPUFAs might hamper their protective effects, including a concerted action of either withdrawing pro-inflammatory eicosanoids or incrementing anti-inflammatory eicosanoids. Eicosanoids and pro-resolving lipid profiles have been recently correlated with survival and clinical outcome in sepsis^34^.

The multivariate models showed that less change in plasmalogen concentrations (plasmenylcholines PC ae C44:6, PC ae 40:2, PC ae 40:5, plasmanylcholine PC ae 38:0), in lysoPC species (e.g. saturated lysoPC C16:0, C18:0), and in fatty acids in combination with larger increment of alanine were associated with non-responsiveness (Fig. 5 and Table 7). Plasmalogens serve as endogenous antioxidants, mediators of membrane structure and dynamics, storage for polyunsaturated fatty acids and lipid mediators^35^. Raising plasmalogen levels protects human endothelial cells during hypoxia^36^. Reduced plasmalogens abundance in NR might reflect an increased oxidative imbalance probably due to an excessive systemic inflammatory response with a resulting high level of oxidative stress. A low plasmalogen level has been reported as a surrogate marker of oxidative stress in elderly septic patients^37^. Furthermore, an exaggerated systemic inflammatory response in NR would be in accordance with the high levels of kynurenine, supporting the role of an accelerated tryptophan catabolism along the kynurenine pathway in sepsis outcome^14, 38^.

A novelty of this study is the emerging role of alanine. Alanine is a gluconeogenic amino acid and plays a key role in the glucose-alanine cycle, a series of reactions in which amino groups and carbons from muscle are transported to the liver. When muscles degrade amino acids for energy needs, the resulting nitrogen is transaminated to pyruvate to form alanine. This is done by the enzyme alanine transaminase, which converts L-glutamate and pyruvate into α-ketoglutarate and L-alanine. The resulting L-alanine is shuttled to the liver where the nitrogen enters the urea cycle and the pyruvate is used to make glucose. Enhanced elaboration of glucose by the liver (hepatic gluconeogenesis) is a prominent feature of the solid organ response to injury and provides fuel to the cellular elements of the inflammatory response. Indeed, the high respiratory exchange ratio for carbohydrate oxidation is the proof that fat oxidation requires more oxygen than carbohydrate oxidation to produce ATP, making glucose more efficient as a fuel source. In this regard, it is well known in sepsis that circulating cytokines and catecholamines cause a shift in metabolism towards the stress state: activation of glycogenolysis and hepatic gluconeogenesis, activation of hepatic lipolysis, increase in muscle protein catabolism and high production of lactate level^39^.

The higher plasma alanine in NR may be a sign of lower hepatic capacity for conversion of alanine to glucose. The higher levels of pyruvic acid and lactic acid in NR patients at T2 (Fig. 1) seem to further support this interpretation.

We recovered also the data of liver functionality markers from the medical records of our patients (Supplemental Table S4 and Supplemental Figures S6 and S7). The values at the two time points of aspartate transaminase (ASAT or AspAT or AAT), alanine transaminase (ALAT or ALT), alkaline phosphatase (AP or ALP), gamma-gamma transaminases (gGT), albumin and total bilirubin were not significantly different between the two groups. Only one patient in R group and one patient in NR group were diagnosed as moderate/severe liver dysfunction at the enrollment. The inflammatory response affects organs functionality, liver included, and the high variability in the values distribution can be easily explained by the shock condition and by antibiotics therapy.

Our hypothesis is that alanine plays a role in the energy metabolism and this pathway could be affected by liver dysfunctionality, but this is a further factor to be investigated in *ad-hoc* clinical trial.

The results presented here highlight biological pathways that could have a clinical impact on septic shock progression and management. Animal experiments are now in progress to understand the time course of metabolome change in this condition better.

We are aware of the risk of overfitting the classifier model to our limited set of subjects in this investigation, despite attempts to minimize these effects with the statistical methods used. Furthermore, these analyses could not take into account all the possible confounding factors such as different renal and hepatic functions, type of nutrition (parental or enteral), and latent insulin resistance.

## Conclusion

In conclusion, the data presented here reinforce the emerging evidence that lipidome alterations are important in the individual patient’s response to infection. Changes in the levels of metabolites over time can distinguish a positive response to therapy. Under conditions of severe inflammatory stress and subsequent elevation of PLA2 enzymes activity, elevation of circulating levels of lysoPCs may promote the consequent inhibition of PLA2 enzymes, thus favoring cytoprotection. The emerging role of alanine suggests a different approach for monitoring hepatic function, which will be more specific than bilirubin. Further studies should investigate whether such metabolic dysregulation could be exploited for a more effective targeted therapy.

## Material and Methods

### Study design, patients and clinical data

This is an ancillary study from the multicenter prospective observational trial named Shockomics (see ClinicalTrials.gov Identifier NCT02141607). Details of the protocol are described in the work of Aletti *et al*.^40^. The study was approved by the Geneva regional research ethics committee (*Commission cantonale d'éthique de la recherché*, President Prof. Bernhard Hirschel, study number 14–041).

Between October 2014 and December 2015, patients admitted with septic shock to the 38-bed mixed ICU of Geneva University Hospital were screened for inclusion criteria according to researchers’ availability. We included adults (>18 years old) with an admission SOFA score ≥6 and arterial lactate ≥2 mmol/l. Patients with a high risk of death within the first 24 hours after admission, systemic immunosuppression, hematological diseases, metastatic cancer, pre-existing dialysis, decompensated cirrhosis or who had received more than 4 units of red blood cells or any fresh frozen plasma before ICU admission were excluded^40^. Informed consent was obtained from patients or proxies.

Patients were managed by the clinical care team according to international guidelines^41^.

Blood samples collected within 16 hours of ICU admission (T1, acute-phase) and 48 hours after enrollment (T2, post-resuscitation phase) were processed for metabolomics analysis^40^.

We used the SOFA score to classify patients in two groups according to their responsiveness to early resuscitation as responders (R) or non-responders (NR). Patients with a SOFA score at T2 higher than 8 and no drop of at least 4 points in their SOFA scores from T1 to T2 (ΔSOFA_T2−T1_) were classified NR.

Survival at 28 days after ICU admission for patients surviving hospital discharge was assessed by consultation of the Geneva Canton death registry and by telephone call to the patient/proxies.

### Untargeted metabolomics by Flow Injection Analysis-Time-of-Flight Mass Spectrometry (FIA-TOF-MS)

#### Plasma samples

Metabolites were extracted by adding four volumes of cold methanol to the plasma sample (10 μL); samples were vortexed and incubated at −20 °C for 1 h. They were then centrifuged 10 min at 14,000 × g, and the supernatant was collected, dried in a SpeedVac and resuspended in 50 μL of 0.1% formic acid^42^. A portion (15 μL) of the extract was analyzed by mass spectrometry.

#### Flow Injection-TOF MS/MS

Analysis was done on an Agilent 1290 infinity Series coupled to an Agilent 6550 iFunnel Q-TOF mass spectrometer (Agilent, Santa Clara, CA) equipped with an electrospray source operated in negative and positive mode. The flow rate was 150 μL/min of mobile phase consisting of isopropanol/water (60:40, v/v) buffered with 5 mM ammonium at pH 9 for negative mode and methanol/water (60:40, v/v) with 0.1% formic acid at pH 3 for positive mode. Reference masses for internal calibration were used in continuous infusion during the analysis (m/z 121.050873, 922.009798 for positive and m/z 11.9856, 1033.9881 for negative ionization). Mass spectra were recorded from m/z 50 to 1100. Source temperature was set at 320 °C with 15 L/min drying gas and nebulizer pressure of 35 psig. Fragmentor, skimmer, and octopole voltages were set to 175, 65, and 750 V, respectively. MS/MS fragmentation patterns of statistically significant features were collected and used to confirmed metabolite identity.

#### MS Data Processing

All steps of data processing and analysis were done with Matlab R2016a (The Mathworks, Natick) using in-house developed script following the workflow proposed by Fuhrer^12^. Centroid m/z lists were exported to csv format. Briefly, in this procedure, we applied a cut-off to filter peaks of less than 500 ion counts for negative and 1000 ion counts for positive ionization to avoid detection of background noise. Centroid m/z lists from different samples were merged to a single matrix by binning the accurate centroid masses within the tolerance given by the instrument resolution (about 10 ppm). The output m x n matrix contains the m peak intensities of each mass for the n analyzed samples. Because mass axis calibration is applied online during acquisition, no m/z correction was applied during processing to correct for potential drifts.

#### Metabolite identification

A total of 14001 and 2190 metabolite masses were measured as peak intensities in positive and negative ion mode respectively. Given the large number of masses measured, we ran preliminary statistical analyses to select only the most significant ones for subsequent metabolite identification. Details of the statistical workflow are reported in Supplemental methods. Significant metabolic species were then identified by database searches (METLIN, http://metlin.scripps.edu; HMBD, http://www.hmdb.ca/) in positive and negative ionization (Table S4).

### Targeted plasma metabolomics analysis

A targeted quantitative approach using a combined direct flow injection and liquid chromatography (LC) tandem mass spectrometry (MS/MS) assay (AbsoluteIDQ 180 kit, Biocrates, Innsbruck, Austria) was applied for the metabolomics analysis to EDTA-plasma samples stored at −80 °C, as previously reported by our group^7^. The method of AbsoluteIDQ p180 kit has been proved in conformance with the FDA Guideline ‘Guidance for Industry—Bioanalytical Method Validation’, which implies proof of reproducibility within a given error range. The method combines derivatization and extraction of analytes with the selective mass-spectrometric detection using multiple reaction monitoring (MRM) pairs. Isotope-labeled internal standards are integrated into the platform for metabolite absolute quantification. This strategy allows simultaneous quantification of 186 metabolites (40 amino acids and biogenic amines, 40 acylcarnitines, 90 glycerophospholipids, 15 sphingomyelins, 1 monosaccharide). For data preprocessing, analytical specification and statistical analyses refer to Supplemental Methods.

A metabolite was excluded from further analysis if its concentration did not meet all the following criteria: (1) fewer than 20% of missing values (non-detectable peak) for each quantified metabolite in each experimental group (2) 50% of all sample concentrations for the metabolite had to be above the limit of detection (LOD)^7^. In total, 130 of the 186 metabolites were used for statistical analysis. All the measurable metabolites are listed in Supplemental Table S5.

### Determination of sPLA2-IIA levels

sPLA2-IIA levels in plasma were detected by using the sPLA2-IIA (human type IIA) ELISA kit (Cayman Chemical, USA) according to manufacturer’s instructions. Concentrations in plasma were tested in duplicate and determined against standard curves at wavelength of 450 nm (Tecan Infinite M200 Plate Reader, Tecan Trading AG, Switzerland).

### Statistical analyses

For those species identified by untargeted metabolomics and also quantified by targeted approach, we verified that their peak intensities and concentrations were correlated using the Spearman correlation analysis (see Supplemental Figure S3). For the identified metabolites, we examined the ability to separate the two groups of each metabolite individually by computing the area under the ROC curve, applying leave-one-out cross-validation (CV) (Supplemental Figure S4). Details of the statistical analysis for the comparisons of peak intensities are given in the Supplemental Methods.

We compared the metabolite concentrations measured by the targeted approach at T1 and T2 of the R and NR groups by the Wilcoxon rank-sum test. Changes in metabolite concentrations from T1 to T2 were compared separately for the R and NR groups by the Wilcoxon signed-rank test. Finally, for each metabolite, we compared the time-trend changes in metabolite concentrations (i.e. ∆ = T2 − T1) between the two groups by the Wilcoxon rank sum test. To deal with the large number of statistical comparisons, we also computed the false discovery rate (FDR), assessed after the bootstrapping procedure. The sample size was increased from 14 to 20 subjects for the R group and from 7 to 10 for NR by bootstrapping with replacement, for a total of 30 observations. The bootstrapping procedure was only used for the FDR assessment. Results were considered significant when p < 0.05 and FDR < 0.15.

For the peak intensities, we also examined the ability to separate the two groups of each metabolite individually by computing the area under the ROC curve, applying leave-one-out cross-validation (CV) (Supplemental Figure S4).

Finally, we compared sPLA2-IIA plasma levels between R and NR by means of Wilcoxon rank-sum test both at T1 and T2 and for the ∆ = T2 − T1. We also evaluated changes in sPLA2-IIA concentrations from T1 to T2 within the same group applying the Wilcoxon signed-rank test.

### Multivariate analyses

#### Targeted metabolomics analysis

The aim of our model was to classify NR patients. We built the model on metabolite concentration changes from T1 to T2 (Δ = T2 − T1). The concentrations are highly correlated and, when the number of observations (21 patients) is much lower than the number of features (130 metabolites) as in our case, one must filter and reduce the number of features entering the model.

We adopted the method proposed by Peng *et al*.^43^, which is the minimal-redundancy-maximal-relevance (mRMR). This algorithm sorts the features according to their relevance to the outcome (maximum relevance criterion) and their redundancy (minimum redundancy criterion) in relation to the other variables. The ranking is based on the mutual information between the outcome and each feature and on the mutual information between each couple of features. We discretized the features distribution according to the interquartile range as suggested by Peng *et al*.^43^ in order to apply the mRMR algorithm.

Multivariate analysis was done using the Elastic Net technique^44^, a shrinkage regression method effective in case of several highly correlated variables. It performs continuous variable selection, causing some of the regression coefficients to be exactly zero, thus reducing the variance of the regression estimates by eliminating redundant features. The subset of variables corresponding to non-zero coefficients can be considered as the variables mainly associated with the outcome. The elastic net technique can be used with any linear regression model. Since we have a binary outcome (R/NR), we applied the logistic regression.

We considered the first 10, 20 and 30 metabolites ranked by mRMR to build three different classification models. The dataset was divided into a training and test set as two third and one third of the observations, respectively. Data were normalized (Z score normalization) before performing the elastic net. We adopted two strategies to further select a smaller subset of features. We performed 50 times an elastic net logistic model using a logit function to fit the training set data. We considered a binary classification (R = 0, NR = 1) and the output of the model is a value between 0 and 1, which represents a sort of probability. We then selected the coefficients of the model with the minimal deviance. We also applied another strategy, we used the shrinkage parameter λ, corresponding to the model with the minimal deviance, to fit another elastic net model and to obtain the coefficients of the logistic regression. In both cases, the models were then evaluated on the testing set and the performance was assessed by the number of correct imputations.

Linear Discriminant Analysis (LDA) and Partial Least Squares Discriminant Analysis (PLS-DA) were also implemented. More precisely, LDA was performed on the first 10 ranked metabolites and the coefficients for the linear boundary between the first and second classes were retrieved. PLS-DA was performed both on the first 10 and 20 ranked metabolites, considering 3 PLS components. Since the groups are unbalanced, the data matrix was weighted centered in order to avoid having a decision boundary shifted towards the most numerous group. The variable importance in projection (VIP) scores, which represent the weights of each feature in PLS-DA model, and the coefficients of LDA were compared to those of logistic regression.

The performance of the classification models was evaluated by the number of correct imputations.

#### Integration of data from targeted and untargeted analysis

We built an integrated model by using the concentration of the metabolites and the peak intensities of the species identified by untargeted analysis. We must precise that for those metabolites quantified also in the targeted approach (i.e. acetylcarnitine, tyrosine and histidine), we used the concentration values instead of peak intensities as they are more reliable. Therefore, the metabolites identified from untargeted approach and used for these analyses were: acetylcarnitine, pyruvic acid, lactic acid, stearic acid, kynuramine, citric acid, myristic acid, palmitoleic acid, palmitic acid, oleic acid, tyrosine, histidine. We built the model on the changes from T1 to T2 (Δ = T2 − T1). Untargeted metabolomics data (10 features in total) were then combined with the first 20 ranked metabolites from targeted analysis. We considered all the 30 features and we performed again the mRMR algorithm to find the first 10, 20 and 30 ranked features. The classification models were built using the regularized logistic regression on normalized data as described in the previous paragraph. LDA and PLS-DA were also performed as stated above.

## Electronic supplementary material

Supplementary material

Table S2
