# Supplementary material for: Characterization of a metabolomic profile associated with responsiveness to therapy in the acute phase of septic shock
Source: Sci Rep. 2017 Aug 29;7:9748. doi: 10.1038/s41598-017-09619-x (PMC5575075; doi:10.1038/s41598-017-09619-x)
Supplement: Supplementary file 1 — Supplementary material [file 41598_2017_9619_MOESM1_ESM.doc]

**Supplemental Information**

**Characterization of a metabolomic profile associated with responsiveness to therapy in the acute phase of septic shock**

Alice Cambiaghi^1^, Bernardo Bollen Pinto^2^, Laura Brunelli^3^, Francesca Falcetta^3^, Federico Aletti^1^, Karim Bendjelid^2^, Roberta Pastorelli^3^*, Manuela Ferrario^1^*

* equally contributed to the work

^1^ Politecnico di Milano, Milan, Italy

^2^ Hôpitaux Universitaires de Genève, Genève, Switzerland-

^3^ IRCCS-Istituto di Ricerche Farmacologiche Mario Negri, Milan, Italy

**Index**

**Supplemental Methods**

**Supplemental Table S1.** Significant metabolites identified at T1 (targeted metabolomics)

**Supplemental Table S2.** Significantly molecular species identified by database search HMBD. (untargeted metabolomics) see provisional excel table.

**Supplemental Table S3**. List of the measurable metabolites using the Biocrates Absolute IDQ p180 kit.

**Supplemental Table S4.** Markers of hepatic functionality at the two time points T1 and T2.

**Supplemental Figure S1**. Significant metabolite concentrations (μM) in responsive (R) and non-responsive (NR) groups at T1.

**Supplemental Figure S2.** AUC analyses for targeted metabolomics.

**Supplemental Figure S3.** Spearman correlation between concentrations and peak intensities of the same species quantified with both approaches (targeted and untargeted respectively).

**Supplemental Figure S4.** AUC analyses for untargeted metabolomics.

**Supplemental Figure S5.** Graph of fold change (D7/D1) in the plasma levels of the most abundant lysoPCs versus sPLA2-IIa in all the patients.

**Supplemental Figure S6.** Time trend of markers of hepatic functionality

**Supplemental Figure S7.** Plasma ALAT (alanine transaminase) levels (U/L) in responsive (R) and non-responsive (NR) patients at T1 and T2.

**Methods**

**Untargeted metabolomics (FIA-TOF-MS).**

*Details on Statistical Analysis*

A total of 14001 and 2190 metabolite masses were measured as peak intensities in positive and negative ion mode respectively. Given the high number of masses measured, we performed preliminary statistical analyses in order to select only the most significant ones for the successive metabolite identification

Firstly, we tested the presence or absence of the species in the two groups, i.e. if the incidence of the peaks at each mass-to-charge ratio (m/z) is different between the groups at T1 and at T2. We constructed contingency tables for each m/z by counting the number of patients in R and NR group having such ion detected (i.e. above the limit of detection) and we applied the Fisher Exact Test by considering the data at T1 and T2 separately. We constructed also contingency tables for each m/z by counting the number of patients having such ion detected at T1 and at T2 and we applied the McNemar test to test whether the incidence of detected masses change from T1 to T2. In positive ion mode, 63 masses at T1 and 172 at T2 have a statistically significant different incidence (pval<0.05) between R and NR, whereas in negative ion mode 8 masses at T1 and 20 at T2. McNemar test was significant (pval<0.05) for 653 and 119 masses in positive and negative mode respectively (results not shown).

As second step, we compared the peak intensities distributions. Unpaired and paired univariate analysis were performed by means of Wilcoxon rank-sum test and by Wilcoxon signed-rank test respectively. For the univariate analysis, only masses for which peaks were detected in more than 5 pts in R group and in more than 3 pts in NR, were considered.

To overcome the problem of the large number of statistical comparisons, in all analyses, the calculation of the false discovery rate (FDR) was applied to the p-values obtained from the tests. Results were considered statistically significant when p<0.05 and FDR <0.15. 25 and 79 masses were significantly different between R and NR at T1 and T2 respectively in positive ion mode; 10 and 19 masses in negative ion mode (Wilcoxon Test, p<0.05). As for the paired analysis (T1 vs T2 within the same group), in positive ion mode, 119 and 48 masses significantly changed from T1 to T2 in R and NR respectively; in negative ion mode 50 and 41. This information was used for the successive analyses for metabolites identification.

*Details on Metabolite identification*

For metabolite identification, the statistically significant m/z values were used for batch searches on metabolomics databases. Metabolic species were identified matching the experimental accurate mass and tandem mass spectra (MS/MS) in positive and negative ionization with those available in metabolomic databases (METLIN and HMDB). Only positively and negatively charged forms of the molecule ([M+H]^+^ or [M-H]^-^) and not additional variants of ions were considered for metabolite identification by means of databases. We did not pursue to identify complex lipids because of the need of internal standards for lipid classes for their unambiguous identification.

Significantly metabolic species identified by database searches and their peak intensities for each sample are listed in Supplemental **Table S4.**

It should be noted that a given molecule may be represented by several different features, such as naturally occurring components of its isotopic cluster or non-specific adduct ions. Several analytes were detected only in positive mode, while others were observed only in the negative ion mode.

**Targeted metabolomics**

*Absolute Metabolite profiling and filtering criteria*

Targeted metabolomics analysis of plasma samples from study subjects was performed using the Biocrates AbsoluteIDQTM p180 kit (Biocrates Life Science AG, Innskruck, Austria). This validated targeted assay allows for simultaneous detection and quantification of metabolites in biological samples in a high-throughput manner. The metabolite extracts were processed following the instructions by the manufacturer and analyzed on a triple-quadropole mass spectrometer (AB SCIEX triple-quad 5500) operating in the multiple reaction monitoring (MRM-MS) mode. The assay is based on PITC (phenylisothiocyanate)-derivatization in the presence of internal standards for the analysis of aminoacids and biogenic amines resolved and quantified by liquid chromatography- tandem mass spectrometry (LC-MS/MS) using scheduled MRMs. Subsequent flow injection analysis tandem mass spectrometry (FIA-MS/MS) was performed to analyze acylcarnitines, glycerophospholipids, hexose. MRM detection was used for quantification applying spectra parsing algorithm integrated into the MetIQ software (Biocrates Life Science AG, Innskruck, Austria). Concentrations were calculated and evaluated by comparing measured analytes in a defined extracted ion count section to those of specific labeled internal standards or non-labeled ones, provided by the kit. The measurements are made in a 96-well format. Seven calibration standards, five quality control samples, three zero samples (methanol) and one blank (solvents) are integrated into the plate. The limit of detection for the individual metabolites is set three times the value of the “zero samples”. The average coefficient of variation of the metabolites among the biological replicates was 30%. This variation is the sum of biological and technical variation. Based on the five quality controls (QCs) included in the mass spectrometric analysis to monitor the instrumental performances and evaluate the quality of the data, the CV was below 15% (technical variation). For glycerophospholipids, the precise position of the double bonds and the distribution of the carbon atoms in different fatty acid side chains cannot be determined with this technology. Consequently, the detected MRM signal is a sum of several isobaric/isomeric lipds. For example, according to LIPID MAPS database ([www.lipidmaps.org](http://www.lipidmaps.org/)) the signal of PCaa C36:6 can arise from at least 15 different lipid species that have different fatty acid composition (e-g. PC 16:1/20:5 versus PC 18:4/18:2, various position of fatty acid sn-1/sn-2 (e.g. PC 18:4/18:2 versus PC 18:2/18:4) and different double bond positions and stereochemistry in those fatty acid chains (e.g. PC(18:4(6Z,)Z,12Z,15Z)/18:2(9Z,12Z) versus PC (18:4(9E,11E,13E,15E)/18:2(9z,12Z)).

Lipid side-chain composition is abbreviated as Cx:y, where x denotes the number of carbons in the side chain and y the number of double bonds. The nature of fatty acids linkage is expressed as aa for diacyl or ae for acyl-alkyl. For example, PCaaC32:1 denotes diacyl-phosphatidylcholine with 32 carbons in the two fatty acids side chains and a single double bond in one of them. The list of all the measurable metabolites is provided in supplemental **table S5.**

|  | **R** | **NR** | **R vs NR** | **pValue** |
| --- | --- | --- | --- | --- |
| **lysoPC a C18:2** | 1.215 (1.050, 1.380) | 0.758 (0.572, 0.990) | ↑ | 0,040 |
| **PC ae C38:5** | 12.90 (11.400, 14.400) | 16.20 (13.900,18.875) | ↓ | 0,011 |
| **PC ae C40:5** | 2.040 (1.770, 2.370) | 2.66 ( 2.395, 2.767) | ↓ | 0,040 |
| **C2 (acetylcanitine)** | 5.790 (3.650, 7.870) | 10.90 (6.872,14.775) | ↓ | 0,040 |

**Table S1 -** Significant metabolites identified by Wilcoxon signed-rank test (pval<0.05 and FDR <0.15) at T1 for the targeted approach. Concentration values are presented as median, 25th and 75th percentile. The arrows indicate if the mass in R group is lower (↓) or higher (↑) with respect to NR group.

**Table S2**. Significantly metabolic species identified by database searches (METLIN and HMBD). The following are reported: peak ID, molecular weight (MW) in kDa, metabolite name, HMBD entry, peak intensity in each sample. Bold metabolites: metabolites whose identification was based on MS^n^ spectra matching whit authentic standard present in HMBD metabolomic database. (See additional excel file)

**Table S3**. List of the measurable metabolites using the Biocrates Absolute IDQ p180 kit

Aa, acyl-acyl; ae, acyl-alkyl; a, lyso; Cx:y, where x is the number of carbons in the fatty acid side chain; y is the number of double bonds in the fatty acid side chain; DC, decarboxyl; M methyl; OH, hydroxyl; PC, phosphatidylcholine; SM, sphingomyeline

| **mETABOLITE CLASS** | **nUMBER** | **mETABOLITE NAME OR ABBREVIATION** | **Biological relevance**  **(SELECTED EXAMPLES)** |
| --- | --- | --- | --- |
| **Amino acids** | 21 | Alanine, arginine, aspartate, citrulline, glutamine, glutamate, glycine, histidine, isoleucine, leucine, lysine, methionine, ornithine, phenylalanine, proline, serine, threonine, tryptophan, tyrosine, valine | Amino acid metabolism, urea cycle, activity of gluconeogenesis and glycolysis, insulin sensitivity, neurotransmitter metabolism, oxidative stress |
| **Carnitine** | 1 | C0 | Energy metabolism, fatty acid transport and mitochondrial fatty acid oxidation, ketosis, oxidative stress, mitochondrial membrane damage |
| **Acylcarnitine** | 39 | C2, C3, C3:1, C3-OH, C4, C4:1, C4-OH, C5, C5:1, C5:1-DC, C5-DC, C5-M-DC, C5-OH, C6, C6:1, C7-DC, C8, C9, C10, C10:1, C10:2, C12, C12-DC, C14, C14:1, C14:1-OH, C14:2, C14:2-OH, C16, C16:1, C16:1-OH, C16:2, C16:2-OH, C16-OH, C18, C18:1, C18:1-OH, C18:2 |
| **Biogenic amines** | 19 | Acetylornithine, asymmetric dimethylarginine, total dimethylarginine, alpha-aminoadipic acid, carnosine, creatinine, histamine, kynurenine, methionine sulfoxide, nitrotyrosine, hydroxyproline, phenylethylamine, putrescine, sarcosine, serotonin, spermidine, spermine, taurine | Neurological disorders, cell proliferation, cell cycle progression, DNA stability, oxidative stress |
| **Lyso-phosphatidylcholines** | 14 | lysoPC a C14:0/C16:0/C16:1/C17:0/C18:0/C18:1/C18:2/C20:3/C20:4/C26:0/C26:1/C28:0/C28:1 | Degradation of phospholipids, membrane damage, signaling cascades, fatty acid profile |
| **Diacyl-phosphatidylcholines** | 38 | PC aa C24:0/C26:0/C28:1/C30:0/C30:2/C32:0/C32:1/C32:2/C32:3/C34:1/C32:2/C34:3/C32:4/C36:0/C36:1/C36:2/C36:3/C36:4/C36:5/C36:6/C38:0/C38:1/C38:3/C38:4/C38:5/C38:6/C40:1/C40:2/C40:3/C40:4/C40:5/C40:6/C42:0/C42:1/C42:2/C42:4/C42:5/C42:6 | Dyslipidemia, membrane composition and damage, fatty acid profile, activity of desaturases |
| **Acyl-alkyl-phosphatidylcholine** | 38 | PC ae C30:0/C30:2/C32:1/C32:2/C34:0/C34:1/C34:2/C34:3/C36:0/C36:1/C36:2/C36:3/C36:4/C36:5/C38:0/C38:1/C38:2/C38:3/C38:4/C38:5/C38:6/C40:1/C40:2/C40:3/C40:4/C40:5/C40:6/C42:0/C42:1/C42:2/C42:3/C42:4/C42:5/C44:3/C44:4/C44:5/C44:6 |
| **Sphingomyelins** | 15 | SM (OH) C14:1, SM C16:0, SM C16:1, SM C16:1, SM C18:0, SM C18:1, SM C20:2, SM C22:3, SM (OH) C22:1, SM (OH) C22:2, SM C24:0, SM C24:1, SM (OH) C24:1, SM C26:0, SM C26:1 | Signaling cascades, membrane damage (eg. neurodegeneration) |
| **Hexose** | 1 | H1 | Carbohydrate metabolism |
| **Total** | 186 |  |  |

|  | **R** | | **NR** | |
| --- | --- | --- | --- | --- |
|  | **T1** | **T2** | **T1** | **T2** |
| **Albumin (g/l)** | 27.5 (23, 29) | 25 (22, 27.5) | 24 (23.25, 27.5) | 23.5 (21, 26) |
| **ASAT (U/l)** | 47 (3, 65) | 39 (27.75, 48.5) | 55 (41.25, 231) | 72 (36.25, 177.75) |
| **ALAT (U/l)** | 29 (22, 44) | 26 (18.25, 35.25) | 51 (21.5, 163) | 44 (22.25, 148.25) |
| **AP (U/l)** | 73.5 (36, 98) * | 96 (53.25, 116.5) * | 62 (4.5, 129.75) | 62 (3.25, 233) |
| **gGT (U/l)** | 6 (22.75, 94) | 76 (47.5, 128.75) | 68 (3.25, 12.5) | 71.5 (32, 123) |
| **Total bilirubin (μmol/l)** | 2 (11, 27) * | 12.5 ( 7.5, 26.5) * | 19 (13.25, 117.5) | 19 ( 9.5, 93.5) |

**Table S4** - Markers of hepatic functionality in the two groups (R: responsive; NR: not responsive to therapy) at the two time points T1 and T2. Data are presented as median, 25th and 75th percentiles. No significant differences were found between the two groups (p-value >0.05, Wilcoxon rank-sum test).
As for time trend, only PA and total bilirubin significantly changed from T1 to T2 in the R group only (p-value <0.05, Wilcoxon sign-rank test).

ASAT: aspartate transaminase (also known as AspAT or AAT); ALAT: alanine transaminase (also known as ALT); AP: Alkaline phosphatase (also known as ALP); gGT: gamma-gamma transaminases.

**Figure S1** – Target metabolomics. Metabolite whose concentration (μM) is statistically different between responsive (R) and non-responsive (NR) groups at T1 (Wilcoxon rank-sum test p < 0.05, FDR < 0.15). Distributions are shown as box-plot, where the central line is the median concentration, the edges of the box are the 25th and 75th percentiles and the outliers are defined as 1.5 times the interquartile range and highlighted by +.

**Figure S2** – AUC analyses for targeted metabolomics. Only the best 30 metabolites are shown (AUC > 0.5). The ability of separating the two groups of the delta of each metabolite individually was evaluated by computing the area under the ROC curve using the leave-one-out cross-validation (CV) technique. Notice that the performance in classifying the two groups is poor: the average ACU of each metabolites is below 0.8.

**Figure S3** – Spearman correlation between concentrations and peak intensities of the same metabolic species quantified with both approaches (targeted and untargeted respectively). Notice that all species have a significant good correlation (ρ > 0.85 and p-value<10^-5^).

**Figure S4** – AUC analyses for untargeted metabolomics. The ability of separating the two groups of delta in peak intensities of the identified metabolic species individually was evaluated by computing the area under the ROC curve using the leave-one-out cross-validation (CV) technique. Notice that the performance in classifying the two groups is poor: the average ACU of each metabolites is below 0.8, with the only exception of Creatinine.


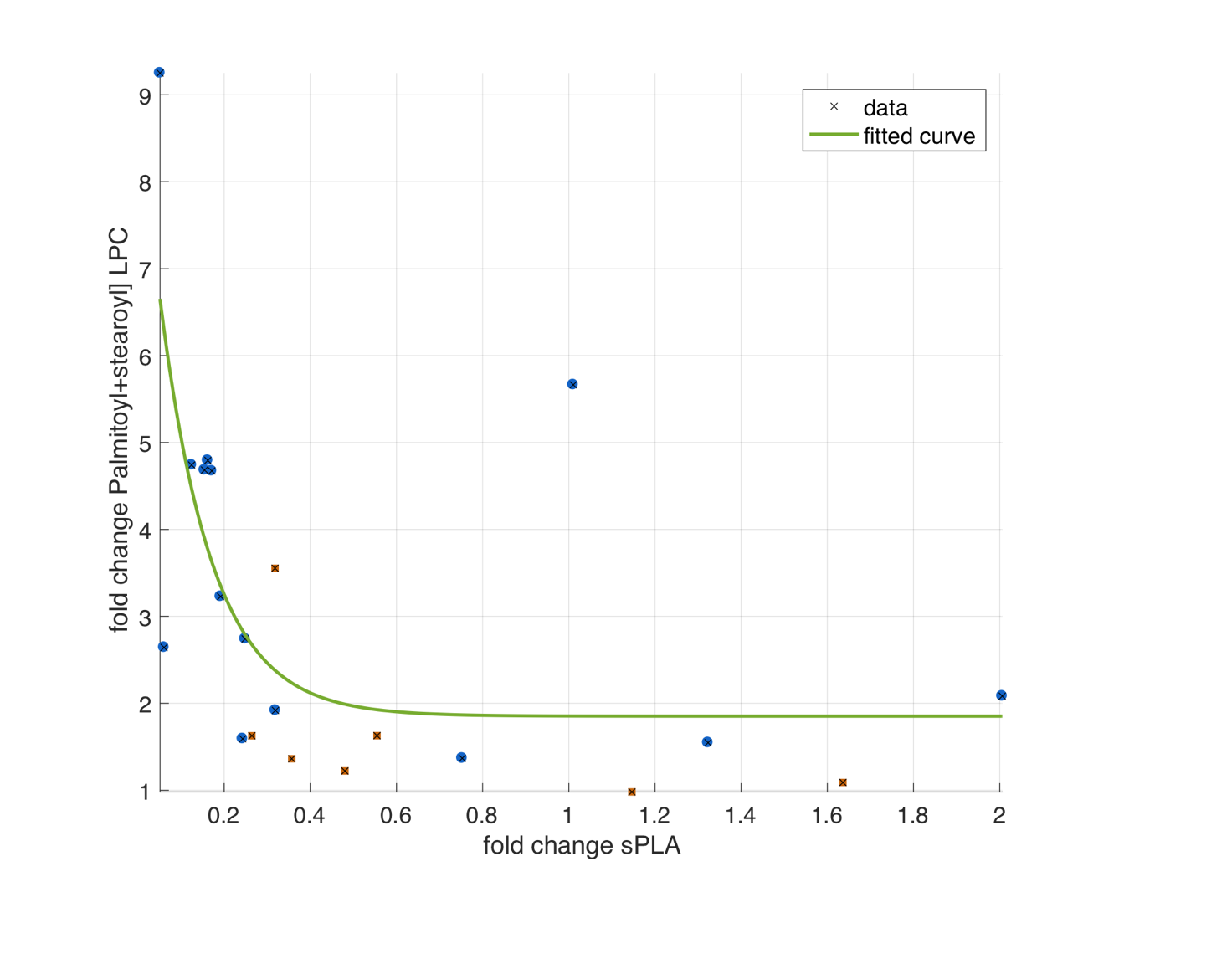


**Figure S5** – Graph of fold change (D7/D1) in the plasma level of the most abundant lysoPCs [palmitoyl (C16:0) and stearoyl (C18:0) lysoPC] versus fold change (D7/D1) in plasma level of sPLA2-IIa in the two groups of patients. We fitted the data with an exponential decay function (green line). Notice that below a certain level (about 0.2) in sPLA2-IIa fold change, the fold change in lysoPC level largely increases (>2).

**Figure S6 –** Time trend of hepatic functionality markers in the two groups (R: responsive; NR: not responsive to therapy). The Wilcoxon sign-rank test was performed to compared the two time points within each group. Significant differences are marked with * (p-value<0.05).

ASAT: aspartate transaminase (also known as AspAT or AAT); ALAT: alanine transaminase (also known as ALT); PA: Alkaline phosphatase (also known as ALP); gGT: gamma-gamma transaminases.

**Figure S7 -**. Plasma ALAT (alanine transaminase) levels (U/L) in responsive (R) and non-responsive (NR) patients at T1 and T2 (panel A) and comparison time trend variation, expressed as delta (Δ=T2 – T1), between the two groups (panel B). Distributions are shown as box-plots where the central line is the median concentration, the edges of the box are the 25th and 75th percentiles and the outliers are defined as 1.5 times the interquartile range and highlighted by +. Although no significant differences were found, we can notice that the trend is similar to the one of sPLA2-IIA (Figure 7), i.e. ALAT levels are higher in NR.
